# Epigenetic variations are more substantial than genetic variations in rapid adaptation of oyster to Pacific oyster mortality syndrome

**DOI:** 10.1126/sciadv.adh8990

**Published:** 2023-09-08

**Authors:** Janan Gawra, Alejandro Valdivieso, Fabrice Roux, Martin Laporte, Julien de Lorgeril, Yannick Gueguen, Mathilde Saccas, Jean-Michel Escoubas, Caroline Montagnani, Delphine Destoumieux-Garzόn, Franck Lagarde, Marc A. Leroy, Philippe Haffner, Bruno Petton, Céline Cosseau, Benjamin Morga, Lionel Dégremont, Guillaume Mitta, Christoph Grunau, Jeremie Vidal-Dupiol

**Affiliations:** ^1^ IHPE, Université de Perpignan Via Domitia, CNRS, Ifremer, Université de Montpellier, Perpignan, France.; ^2^ IHPE, Université de Montpellier, CNRS, Ifremer, Université de Perpignan Via Domitia, Montpellier, France.; ^3^ LIPME, INRAE, CNRS, Université de Toulouse, Castanet-Tolosan, France.; ^4^ Division de l'expertise sur la faune Aquatique, Ministère des Forêts, de la Faune et des Parcs (MFFP), 880 chemin Sainte-Foy, G1S 4X4 Québec, Québec, Canada.; ^5^ Ifremer, IRD, Université de la Nouvelle-Calédonie, Université de La Réunion, ENTROPIE, Nouméa, Nouvelle-Calédonie, France.; ^6^ MARBEC, Université de Montpellier, CNRS, Ifremer, IRD, Sète, France.; ^7^ Université de Brest, Ifremer, CNRS, IRD, LEMAR, F-29280 Plouzané, France.; ^8^ Ifremer, ASIM, Adaptation Santé des Invertébrés Marins, La Tremblade, France.; ^9^ Université de la Polynésie Française, ILM, IRD, Ifremer, F-98719 Tahiti, French Polynesia, France.

## Abstract

Disease emergence is accelerating with global changes. Understanding by which mechanisms host populations can rapidly adapt will be crucial for management practices. Pacific oyster mortality syndrome (POMS) imposes a substantial and recurrent selective pressure on oyster populations, and rapid adaptation may arise through genetics and epigenetics. In this study, we used (epi)genome-wide association mapping to show that oysters differentially exposed to POMS displayed genetic and epigenetic signatures of selection. Consistent with higher resistance to POMS, the genes targeted included many genes in several pathways related to immunity. By combining correlation, DNA methylation quantitative trait loci, and variance partitioning, we revealed that a third of phenotypic variation was explained by interactions between the genetic and epigenetic information, ~14% by the genome, and up to 25% by the epigenome alone. Similar to genetically based adaptation, epigenetic mechanisms notably governing immune responses can contribute substantially to the rapid adaptation of hosts to emerging infectious diseases.

## Teaser

Similar to genetically based adaptation, epigenetic mechanisms can contribute to hosts rapid adaptation to emerging diseases.

## INTRODUCTION

There has been a substantial increase in the emergence of nonhuman pathogens (epizootics) resulting from human-linked activities, including anthropogenic-driven climate change, pollution, habitat fragmentation, overexploitation, local biodiversity impoverishment, and transport of living organisms (*1*–*3*). Some marine epizootics have substantially disturbed ecosystems or social-ecological systems when affecting host species of ecological or economic interest (*4*). Understanding how host populations can adapt rapidly to emerging infectious diseases will be essential for developing effective and ecologically appropriate management practices.

Host-pathogen interactions are characterized by reciprocal selective pressures that both partners impose on each other, and emerging diseases present an opportunity to study rapid selective evolutionary processes. Recent hypotheses propose that the natural phenotypic variation induced by host-pathogen selective pressures could be driven by both genetic and nongenetic components (*5*, *6*). Pacific oyster mortality syndrome (POMS) represents an opportunity to better understand rapid host adaptation to an emerging pathogen. The Pacific oyster (*Crassostrea gigas*) is the most widely farmed oyster worldwide and one of the main marine resources produced by aquaculture. However, since the emergence of the ostreid herpesvirus 1 microvariant (OsHV-1 μVar) in 2008, young oysters (less than 24 months old) living in high density [e.g., oyster farm and wild oyster beds that often co-occur in farming areas (*7*)] face an annual rate of mortality ranging from 40 to 100% worldwide (*8*, *9*).

POMS is a polymicrobial disease, initiated by OsHV-1 μVar infection, which induces lethal bacteremia (*10*). Temperature (*11*) and food availability (*12*) facilitate the establishment and/or development of POMS by altering oyster physiology. Disease susceptibility has a heritable component (*13*–*15*). Genome-wide association (GWA) mapping has revealed that disease resistance has a polygenic architecture (*15*–*17*). However, oyster resistance to POMS is also dependent on oyster life history, such as age (*18*, *19*), and past exposure to pathogen elicitors (*20*, *21*). Exposure to nonpathogenic microbes (*22*) also had an effect, with microbial exposure being associated with epigenetic modifications (i.e., DNA methylation) transgenerationally transmitted to offspring (*22*). These results suggest that natural oyster populations experiencing POMS constitute a useful host-pathogen system for studying the genetic and epigenetic mechanisms underlying rapid adaptation (*23*).

In this study, we identified the genetic and epigenetic bases associated with POMS exposure and their relative contributions to the ongoing adaptation to POMS. To do so, we used a whole-exome capture approach to jointly study one component of the genetic variation (single-nucleotide polymorphism, SNP) and one component of the epigenetic variation (DNA methylation in the CG context, CpG). The genetic and epigenetic bases associated with natural variation in oyster resistance to POMS were detected by carrying out GWA and epigenome-wide association (EWA) mapping, respectively. Subsequent correlation analyses, methylation quantitative trait loci (MethQTL) mapping, and variance partition methods (redundancy analysis, RDA) allowed us to quantify the relative contribution of genetic and epigenetic variations underlying adaptation to POMS.

## RESULTS

### POMS resistance differs between wild oyster populations

**Fig. 1.**
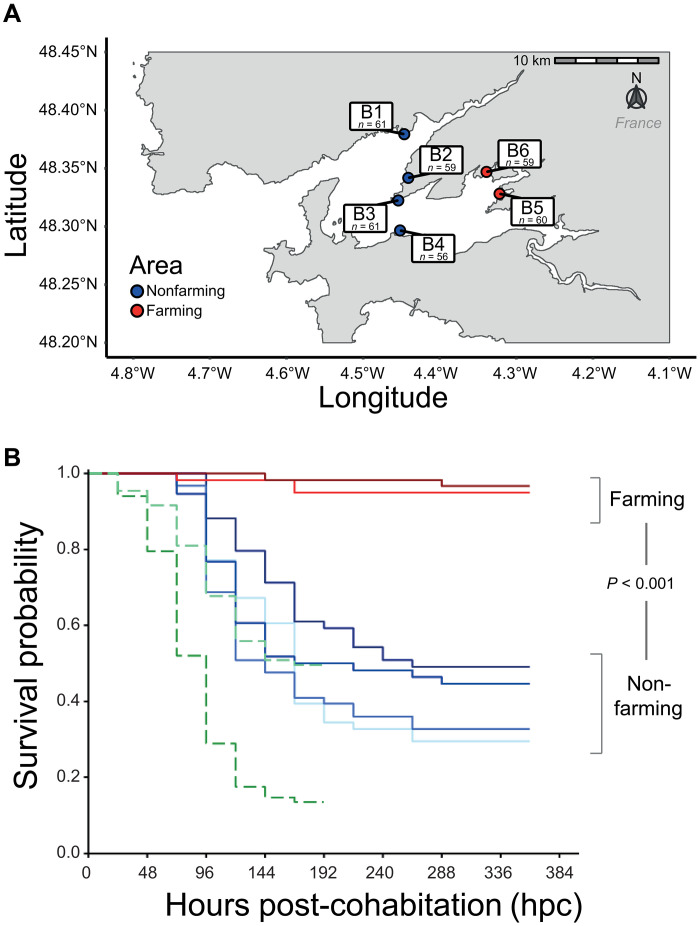
Experimental design and survival after POMS infection. (**A**) Oyster populations sampled in four nonfarming (low density, less than 20 individuals/m^2^ and no POMS reported) and two farming areas (high density with the typical oyster bed of hundreds individuals/m^2^ and annual POMS reported) in the Rade de Brest (France). The *n* represents the number of oysters sampled in each location (total size experiment *N* = 356). (**B**) The Kaplan-Meier survival curves of the donor (dashed line) and recipient oysters (solid line) during the cohabitation experiment. Note that donor oysters were removed from the tanks at 196 hpc.

To phenotype POMS resistance, we sampled four wild oyster populations from a “nonfarming area” (B1 to B4) and two populations from a “farming area” (B5 and B6) in the Rade de Brest, northwest France (Fig. 1A and table S1). The collected oysters were acclimatized in eight experimental tanks and were subjected to experimental infection (randomized complete block design) by cohabitation with infected donor oysters from two oyster families (H12 and NSI) originating from an Ifremer (Institut Français de Recherche pour l'Exploitation de la Mer) hatchery. A lower risk of mortality was detected for the oyster populations from the farming area (B5 and B6) than for the four populations from the nonfarming area (B1 to B4) (log-rank test, *P* < 0.001; table S2). Resistance to disease reached > 94.9% in the farming area populations (Fig. 1B), whereas susceptibility was high (71.5 to 49.1%) in the nonfarming area (Fig. 1B). We coded these phenotypes either as a binary or as a semiquantitative trait (see Material and Methods for details; data S1).

Mortalities among donor oysters (H12 and NSI) started at 24 hours post-cohabitation (hpc). At 192 hpc, the survival rate dropped to 13.5 and 49.5% for H12 and NSI donors, respectively (Fig. 1B). Quantification of OsHV-1 μVar in the experimental tanks showed that viral shedding reached 1755 ± 429 genome copies/μl seawater (mean ± SD) at 24 hpc and peaked at 72 hpc (7185 ± 1856 genome copies/μl). We did not observe any significant difference in the load of virus between the experimental tanks (Kruskal-Wallis, *P* = 0.24; fig. S1). Mortalities in recipient oysters (i.e., oysters from B1 to B6 populations) started at 72 hpc (Fig. 1B) and did not differ significantly among the eight replicate tanks (log-rank test, *P* = 0.61; fig. S2). The farming area oyster populations, which have been subjected to high pathogen pressure, have therefore become resistant to POMS disease, whereas the nonfarming area populations, which have not experienced the disease, were highly susceptible (*24*).

### POMS resistance is associated with immune pathways

We performed an exome-capture experiment using bisulfite-converted DNA to characterize genetic (SNP) and epigenetic (CpG) variation in both susceptible and resistant oysters. In total, 116 susceptible and 130 resistant oysters’ exome were captured and sequenced. On average, sequencing resulted in 0.5 million to 60 million paired-end (PE) reads per sample (mean ± SD = 26 ± 1 millions), and we discarded the six samples that displayed less than 7.8 million PE reads. On average, 59.3 ± 2.7% of PE reads were uniquely mapped to the *C. gigas* genome, and the bisulfite conversion efficiency was 99.6 ± 0.1% (data S2). SNPs and DNA methylation calling resulted in 5,110,093 SNPs and 3,449,600 CpGs for the 240 oysters analyzed. After applying filtering criteria for GWA and EWA mapping, we obtained data for 102 susceptible and 118 resistant oysters, characterized by 214,263 SNPs and 635,201 CpGs (data S2). Analysis of multivariate homogeneity of group dispersions (variances) among the six populations did not reveal any signature of population structure at the genetic and epigenetic levels (fig. S3). A permutational multivariate analysis of variance (PERMANOVA) analysis confirmed this result with no genetic variance (*R*^2^ = 2.3%, *P* = 0.091) and a small percentage of epigenetic variance (*R^2^* = 2.4%, *P* < 0.001) explained by differences among the six populations.

**Fig. 2.**
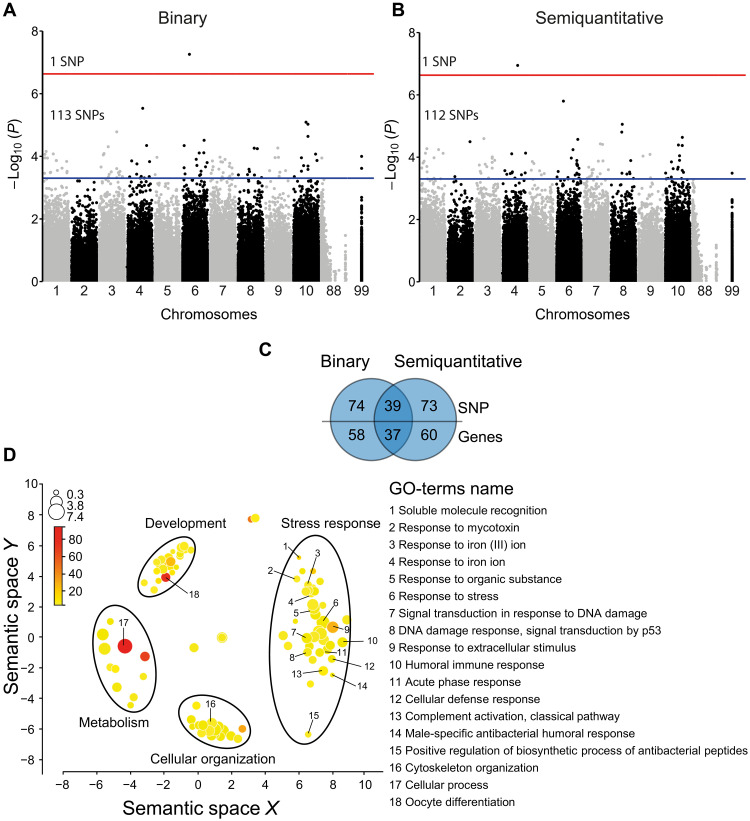
Genetic variation associated (SNPs) with resistant phenotype to POMS. Results of GWA mapping with (**A**) binary phenotype or (**B**) semiquantitative trait of POMS resistance. Chromosomes 88 and 99 correspond to unknown regions in the genome. The red line represents the threshold for an FDR < 0.05 (significant SNPs), and the blue line represents the threshold for a *P* value < 0.0005 (suggestive SNPs). (**C**) Venn diagram comparing the number of SNPs and genes between associations obtained from the binary and semiquantitative phenotype. (**D**) Gene enrichment analysis (GO-terms) of the biological process (BP) from the set of genes displaying significant and suggestive SNPs for the binary and semiquantitative GWA mapping. GO-terms are distributed in multidimensional semantic similarities. The size of the circles (log_10_ size) and the color saturation (log_10_ Fisher’s *P* value) indicate the number of genes represented and the significance value for each GO-term, respectively.

GWA mapping revealed one SNP significantly associated with the binary trait (susceptibility versus resistance): scaffold1832_479264; A>T; *P* = 5.53 × 10^−8^ (Fig. 2A, fig. S4A, and data S3), and one SNP with the semiquantitative trait (survival time expressed in hours of an individual after its exposure to the OsHV-1 μVar): scaffold364_478394; C>T; *P* = 1.13 × 10^−7^ (Fig. 2B, fig. S4B, and data S4). The SNP associated with the binary trait was mapped on chromosome 6 in a gene encoding the SUMO-activating enzyme subunit 2 also known as *UBA2* (CGI_10018487), and the SNP associated with the semiquantitative trait was mapped on chromosome 4 in a gene of unknown function (CGI_10022698). Given the low number of significant SNPs identified and the polygenic architecture of POMS resistance (*15*–*17*), we extended our analysis to suggestive SNPs (*P* < 0.0005; false discovery rate, FDR < 0.9), which led us to the identification of 113 and 112 SNPs associated with the binary and semiquantitative traits, respectively (data S3 and S4). Among these 225 SNPs, 186 of which were nonredundant, 39 were in common, and 74 and 73 were specific to the binary and semiquantitative traits, respectively (Fig. 2C and data S5). The 186 SNPs were located in 155 genes, with 37 genes in common, and 58 and 60 being specific to the binary and semiquantitative traits, respectively (Fig. 2C and data S6).

**Fig. 3.**
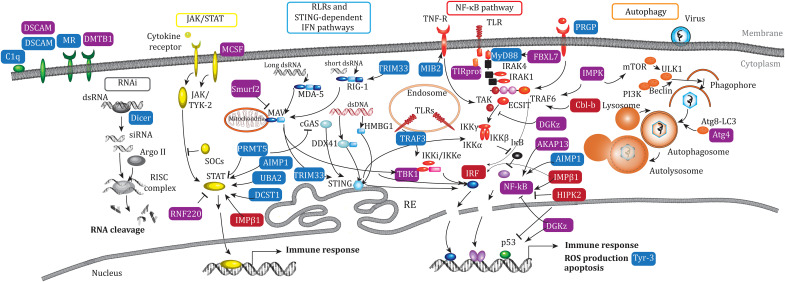
Genes involved in innate immune pathways. Genes involved in innate immune signaling pathways identified with genetic variation only (blue: SNP, 14 genes), epigenetic variation only (red: CpG, 4 genes), and genetic and epigenetic variation (violet: SNP + CpG, 1 gene; MethQTL, 12 genes).

Enrichment analysis of Gene Ontology (GO-terms) revealed the enrichment of four groups of biological process (BP) category: development, metabolism, cellular organization, and stress response (Fig. 2D and data S7). In the last category, we retrieved key stress response BPs, including cellular defense response, humoral immune response, complement activation classical pathway, positive regulation of biosynthetic process of antibacterial peptides, response to extracellular stimulus, signal transduction in response to DNA damage, and DNA damage response (Fig. 2D). In these BPs including immune processes, we identified genes involved in the Janus kinase (JAK)/signal transducer and activator of transcription (STAT) pathway (e.g., protein arginine methyltransferase, *PRMT5*; aminoacyl tRNA synthetase complex interacting multifunctional protein 1, *AIMP1*; *UBA2*, and DC-STAMP domain containing 1,* DCST1*), the STING/RLRs pathway (e.g., tripartite motif containing 33, *TRIM33* and tumor necrosis factor receptor-associated factor 3, *TRAF3*), the Toll-like receptor (TLR)/nuclear factor κB (NF-κB) pathway (e.g., MIB E3 ubiquitin-protein ligase 2, *MIB2*; MYD88 innate immune signal transduction adaptor, *MyD88*; peptidoglycan recognition protein 2, *PRGP*, and TANK binding kinase 1, *TBK1*), the RNA interference (RNAi) pathway (Dicer 1, ribonuclease III, *Dicer*), and pathogen recognition (e.g., proprotein convertase subtilisin/Kexin type 1, *C1q*; protein tyrosine phosphatase receptor type F, *DSCAM*, and mannose binding lectin 2, *MR*; Fig. 3). Thus, resistance to POMS events is associated with genetic variation in multiple genes of key immune pathways.

**Fig. 4.**
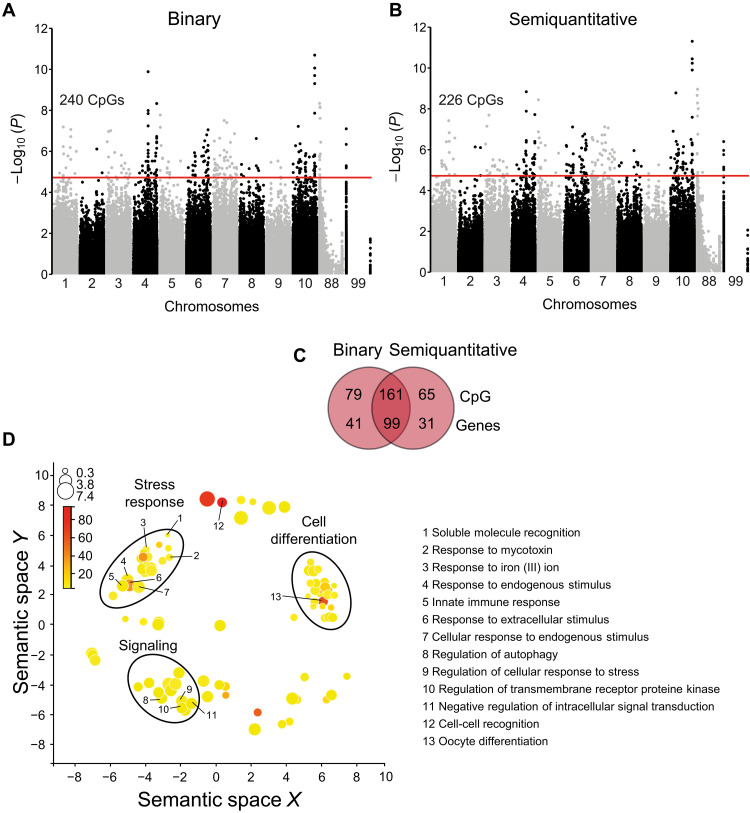
Epigenetic component associated (CpG) with the resistant phenotype to POMS. Results of EWA mapping with (**A**) binary phenotype or (**B**) semiquantitative trait of POMS resistance. Chromosomes 88 and 99 correspond to unknown regions in the genome. The red line represents the threshold for an FDR < 0.05 (significant CpGs). (**C**) Venn diagram comparing the number of CpGs and genes between associations obtained from the binary and semiquantitative phenotype. (**D**) Gene enrichment analysis (GO-terms) of the biological process (BP) from the set of genes displaying significant and suggestive CpGs for the binary and semiquantitative EWA mapping. GO-terms are distributed in multidimensional semantic similarities. The size of the circles (log_10_ size) and the color saturation (log_10_ Fisher’s *P* value) indicate the number of genes represented and the significance value in each GO term, respectively.

To investigate the epigenetic differences between susceptible and resistant oysters, we analyzed the EWA mapping that revealed that the methylation level of 240 (data S8) and 226 (data S9) CpGs were significantly associated with the binary and semiquantitative traits, respectively (Fig. 4, A and B, and fig. S4, C and D). Among these 446 CpGs (305 being nonredundant), 161 were in common, and 79 and 65 CpGs were specific to the binary and the semiquantitative traits, respectively (Fig. 4C and data S10). Among the 305 nonredundant CpGs, 23 and 282 were hypermethylated and hypomethylated, respectively, in the resistant oysters compared to the susceptible oysters. At the gene level, 171 genes displayed at least one differentially methylated CpG, 99 were in common, and 41 and 31 were specific to the binary or semiquantitative traits, respectively (Fig. 4C and data S11).

Enrichment analysis of GO-terms revealed three main groups of BP category: cell differentiation, stress response, and signaling (Fig. 4D and data S12). We retrieved key immune BPs in the stress response and signaling categories, that is, innate immune response, response to endogenous stimulus, response to extracellular stimulus, cellular response to endogenous stimulus, regulation of autophagy, regulation of cellular response to stress, regulation of transmembrane receptor protein kinase, and negative regulation of intracellular signal transduction (Fig. 4D). We identified several immune genes known to be involved in JAK/STAT pathway (e.g., colony stimulating factor 2 receptor subunit alpha, *MCSF*; ring finger protein 220, *RNF220*, and karyopherin subunit beta 1, *IMPβ1*), the STING/RLRs pathway (e.g., SMAD specific E3 ubiquitin protein ligase 2, *Smurt2* and TBK1), the TLR/NF-κB pathway (e.g., toll like Receptor 4, *TIRprot*: F-box and leucine rich repeat protein 7, *FBXL7*; inositol polyphosphate multikinase, *IMPK*; cbl proto-oncogene B, *Cbl-b*; diacylglycerol kinase zeta, *DGKz*; adenosine kinase–associated protein 13, *AKAP13*; AIMP1; IMPβ1; homeodomain interacting protein kinase 2, HIPK2; TBK1; nuclear factor kappa B subunit 1, *NF-κB*, and interferon regulatory factor, *IRF*), pathogen recognition (e.g., deleted In malignant brain tumors 1, *DMTB1*), and the autophagy pathway (IMPK and autophagy related 4C cysteine peptidase, *ATG4C*; Fig. 3A). The results of our EWA mapping therefore revealed that POMS events may have induced and/or selected epigenetic variation in multiple genes including several genes belonging to key immune pathways.

### POMS resistance is associated with genetic and epigenetic variations in common processes

**Fig. 5.**
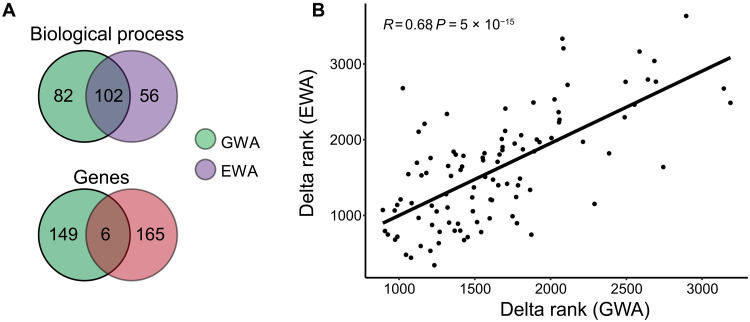
Selection target same biological functions but different genes among genetic and epigenetic variation. (**A**) Venn diagram comparing the GO-terms of biological process (BP) and genes between GWA and EWA mapping. (**B**) Correlation between the delta ranks of the GO-terms significantly enriched from the gene sets obtained with GWA and EWA analysis.

Among the 240 GO-terms enriched in the GWA and EWA mapping, 102 were in common, and 82 and 56 were specific to genetic and epigenetic variation, respectively (Fig. 5A and data S13). The delta rank of the 102 common GO-terms enriched in the GWA and EWA mapping showed a significant and positive correlation (Pearson correlation coefficient *R* = 0.68, *P* < 0.01; Fig. 5B). Of the 320 genes identified in the GWA and EWA mapping, each had at least one significant/suggestive SNP or one significant CpG (data S14), and 149 were specific to genetic variation and 165 to epigenetic variation, with six genes displaying both one significant/suggestive SNP and one significant CpG (Fig. 5A), including TBK1, a major activator of antiviral pathways (Fig. 3). POMS may have therefore led to the selection of genetic variants and the selection or induction of epigenetic variants in different genes, which nonetheless are involved in similar biological functions, in particular, in several genes involved in innate immunity.

### POMS resistance is associated with independent genetic and epigenetic variations

**Fig. 6.**
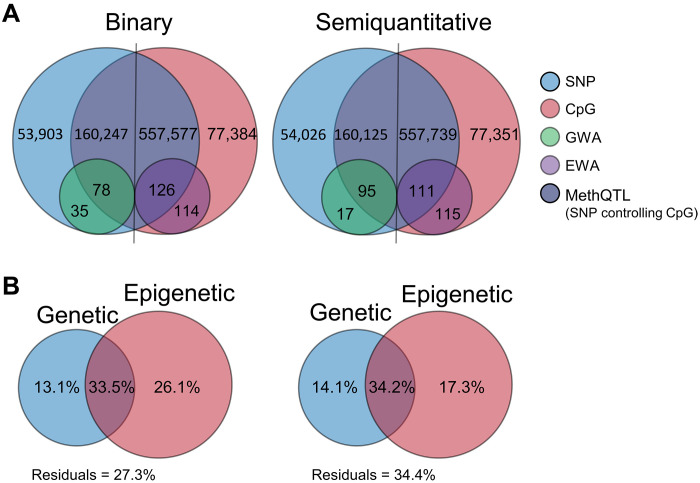
Epigenetic variation explains more phenotypic variation. (**A**) Venn diagrams comparing the results of MethQTL mapping obtained with the binary (left) or semiquantitative (right) phenotype as the covariate. (**B**) Results of variance partition analysis (RDA) to disentangle and weight the portion of phenotypic variation (binary and semiquantitative approaches) explained by the genetic variation, the epigenetic variation, and their interaction.

To quantify the relative contribution of genetic and epigenetic variation to the phenotypic variation, we observed in POMS resistance, tested the relationship between the matrix of pairwise genetic and epigenetic distances, and detected a significant but weak correlation (Mantel statistic ρ = 0.089, *P* = 0.0184). We also used MethQTL mapping, which identified 5,151,194 and 5,152,611 significant SNP-CpG pairs (FDR < 0.05) when using the binary and the semiquantitative trait as a covariate, respectively. Removing redundant SNP-CpG pairs, we identified 160,325 (binary) and 160,220 (semiquantitative) SNPs associated with the DNA methylation rate of 557,703 (binary trait) and 557,850 (semiquantitative trait) CpGs (Fig. 6A and table S3), showing that a large portion (~88%) of the epigenetic variation is associated with genetic variation. However, when we specifically looked for the CpGs significantly associated with POMS resistance (EWA), 126 over 240 (binary) and 111 over 226 (semiquantitative) were identified by MethQTL mapping, thereby suggesting that ~50% of the significant CpGs displayed a DNA methylation rate independent of the DNA sequence (Fig. 6A and table S3).

Variance partition analysis (RDA) showed that genetic and epigenetic variation jointly explained the highest percentage of phenotypic variation, with 33.5 and 34.2% for the binary and semiquantitative traits (Fig. 6B). In addition, epigenetic variation explained a higher proportion of phenotypic variation (binary trait = 26.1% and semiquantitative trait = 17.3%) than genetic variation (binary trait = 13.1% and semiquantitative trait = 14.1%; Fig. 6B).

Thus, most of the genetic and epigenetic variations associated with POMS resistance were correlated with each other. However, some of the genetic and epigenetic variation remains independent, suggesting that changes in the epigenome, independent of genetic changes, have contributed to POMS resistance.

## DISCUSSION

Here, we show that populations of wild oysters exposed to POMS displayed an association that may reflect selection both in their genome (SNPs) and their epigenome (CpG DNA methylation). These signatures of selection, on genetic and epigenetic variation, targeted the same biological processes (e.g., immunity) but acted through different genes. Correlation analysis between genetic and epigenetic variation and MethQTL mapping showed that genetic and epigenetic variations are partially correlated and that genetic variation influences a large proportion of the epigenetic variation (88%). However, ~50% of CpGs significantly associated with oyster resistance are among the 12% of epigenetic variation not associated with genetic variation (MethQTL mapping), suggesting that epigenetic variation can occur independently of genetic variation, at least in our study context. These results were confirmed by RDA, showing that the expressed phenotypic variance was mainly explained (33.5 to 34.2%) by the interaction between genetic and epigenetic variation, a smaller fraction (17.3 to 26.1%) by epigenetic variation alone, and the smallest fraction (13.1 to 14.1%) by genetic variation alone. These results suggest that a host population facing recurrent and strong pathogen selection pressure selects genetic variants and selects or induces epigenetic variants during rapid adaption.

In our study, we took advantage of the differential environmental selective pressure exerted by POMS on wild oyster populations, in farmed areas versus nonfarmed areas, to quantify the interaction and relative effect of genetic and epigenetic variations on phenotypic variation (i.e., resistant versus susceptible). GWA and EWA mapping carried out by grouping the resistant versus susceptible individuals irrespective of their population of origin to avoid the detection of signature linked to (epi)genetic drift enabled us to identify phenotype-genetic/epigenetic associations reflecting a putative selection both in the genome and the epigenome. We quantified the relative effect of the genetic and epigenetic variation using genetic/epigenetic matrix correlation tests (partial Mantel test), MethQTL mapping (control of CpG methylation level by SNPs), and variance partition analysis by RDA. These analyses demonstrated that most genetic and epigenetic variations are linked, given that methylation of ~88% of CpGs was significantly associated with one or more SNPs. In contrast, the remaining 12% of CpGs that were not significantly associated with SNPs, included ~50% of the associations detected by EWA mapping. We can conclude that the adaptive phenotype observed in response to POMS selection may involve both genetic and epigenetic variation, much of which is correlated. However, there is also independent genetic and epigenetic variation significantly associated with the selected phenotype of resistance to POMS.

Significant associations between genetic or epigenetic variation and environmental parameters (e.g., temperature or salinity) have previously been reported in various invertebrates (*25*–*29*), with a significant correlation between genetic and epigenetic variation found in 6 of 14 studies reviewed (*30*). MethQTL mapping revealed that the fraction of epigenetic variation associated with DNA sequence variation (*26*, *28*, *31*) is highly variable, ranging from 2% in the threespine stickleback (*Gasterosteus aculeatus*) (*31*), 3% in the Olympia oyster (*Ostrea lurida*) (*28*), 19% in *Ciona intestinalis* (*26*), 70% in human (*Homo sapiens*) (*32*, *33*), and 88% in this study. Complementary approaches in invertebrates and vertebrates have shown that 27% of interindividual epigenetic variation is genotype dependent (*28*) and 24–35% of the epigenetic variation is explained by additive genetic components (*31*). Our results are consistent with these studies, which, altogether, demonstrate not only that genetic and epigenetic variation are linked but also that a substantial proportion of the epigenetic variation can be independent of the genetic variation, and vice versa, such that each on its own can contribute to an adaptive phenotype.

Variance partition analysis (RDA) further supported this conclusion, showing that 33.5 to 34.2% of the observed phenotypic variation was explained by the interaction between genetic and epigenetic variation, 26.1 to 17.3% by epigenetic variation alone, and 14.1 to 13.1% by genetic variation alone. Similar approaches had been used before to analyze gene expression variation between sister species of whitefish, the Lake whitefish (*Coregonus clupeaformis*) and the European whitefish (*Coregonus lavaretus*), both comprising sympatric benthic and limnetic specialists (*34*). In the work of Rougeux *et al.* (*34*), 46.7% of gene expression variation was explained by the interaction between genetic and epigenetic variation, 4.1% by genetic variation alone, and 2.1% by epigenetic variation alone. The large differences in the relative contribution of genetic and epigenetic variation to phenotypic variation may reflect differences in mechanism acting at the macroevolutionary versus microevolutionary scale. In our study, the selected oysters faced a strong environmental constraint due to an infectious disease that recently emerged (14 years ago), while the whitefish study focused on an evolutionary step that occurred during the Pleistocene (~12,000 years ago) (*34*). At the microevolutionary scale, a population experiencing a strong selection pressure will likely rely on the rapid emergence of phenotypes close to the new phenotypic optimum. Epigenetic variation can occur quickly and is reversible, thereby allowing for a rapid increase of the phenotypic space, whereas the evolution of genetic variation is generally slower and is almost always not reversible. The selected phenotypes originating from epigenetic variation could be then maintained for a longer evolutionary time through genetic assimilation, the latter being incidentally promoted by epigenetically facilitated mutational assimilation.

In our study, the nongenetic related epigenetic variation associated with resistance to POMS may have arisen from environmental exposure or from random epimutations subsequently selected for by POMS. POMS interactions with the oyster immune system have demonstrated that oyster resistance/susceptibility can be influenced by both intrinsic and extrinsic factors (*35*). For example, exposure to a nonpathogenic microbiota during early life (*22*) or exposure to viral mimics (polyinosinic:polycytidylic acid) at the juvenile (3 months old) stage (*21*, *36*) can increase long-term immune competence, both within and across generations. Such environmental exposure induced a significant increase in resistance to POMS. Exposure to microbiota modified DNA methylation patterns (*22*), some of which were transmitted to the subsequent generation, although this second generation was never exposed to the microbiota (*22*). Changes in the epigenome induced by the environment in oysters and the inheritance of DNA methylation patterns have also been demonstrated in two other studies (*28*, *29*). Thus, the environment may induce heritable epigenetic modifications in oysters that may contribute to adaptive phenotypic variation (*22*, *28*, *29*). We hypothesize that, in our study, natural microbial exposure experienced in the field may have facilitated the development of resistance to POMS either directly (environmentally induced modifications) or indirectly (inherited environmentally induced modifications) in the oyster population sampled in farming areas.

Exome capture, sequencing, and downstream analysis performed in this study showed that the signatures of oyster resistance to POMS were carried by both genetic and epigenetic marks, 186 suggestive SNPs and 305 significant CpGs, respectively. The gene ontology enrichment analysis showed among other processes an enrichment of biological processes linked to immunity (Figs. 2D and 4D). Genes involved in these immune processes have been identified (Fig. 3) and participate in the JAK/STAT pathways (*UBA2* and *RNF220*), the RLR/STING pathway (*TRIM33*, *TBK1*, and *IRF*), the NF-κB pathway (*TIRprot*, *NF-κB*, and *MyD88*), RNAi (*DICER*), and autophagy (*ATG4C*), as well as several pathogen recognition receptors, such as *DSCAM*, mannose receptors, *C1q*, and *PRGP* (*37*) , consistent with previous studies demonstrating the polygenic nature of POMS resistance at the genetic level (*15*–*17*, *38*). The POMS resistance phenotype involves antiviral genes and pathways, either constitutively expressed and up-regulated faster in response to POMS in resistant families or environmentally induced (*20*–*22*). These studies demonstrate that many immune system–related genes are involved in the POMS resistance phenotype, but few are common across the oyster families or populations studied here. This polygenic nature of the response provides multiple solutions against the POMS, which is selectively advantageous. It might also illustrate an ongoing adaptation following an infinitesimal-like model. Models of polygenic adaptation after a sudden change in environment, such as a pathogen emergence, predict a two-phase process where the first phase (the rapid phase) leads to the increase in the frequency of “myriad of (epi)alleles” encoding a mean phenotype close to the new optimum (*39*). This rapid phase could be represented by not only the spread of our genetic and epigenetic signatures across genes of several immune pathways but also other biological functions.

The present study showed that in response to the recent emergence of an epizootic disease inducing a strong selective pressure, the oyster population presents heritable phenotypic variants with selective advantages that are associated with genetic and epigenetic variations. While our study confirms the essential role played by genetics and also shows that epigenetic variation is associated with genetic information, we further demonstrated that epigenetic variation can also function independently and, in our case, play a major role in explaining phenotypic variance, with all these mechanisms acting together to rapidly promote the emergence of new adaptive phenotypes.

## MATERIALS AND METHODS

### Oyster sampling strategy: Farming and nonfarming areas

Wild oysters of *C. gigas* (~14 months old) were collected in October 2018 from six natural populations referred to as B1 to B6 located in the Rade de Brest (France). A total of 356 oysters were collected with ~60 individuals per population (Fig. 1A and table S1). Two populations (B5 and B6) were in oyster “farming areas” [natural oyster beds with hundreds individuals/m^2^ collocated with oyster farm; fig. S5; annual POMS events (*24*)], and the four other populations (B1 to B4) were in “nonfarming areas” (natural populations of low density with less than 20 individuals/m^2^ without oyster farm in its vicinity; fig. S5; no POMS events). Length and width of sampled oysters were recorded, but it is important to note that these parameters can vary between oyster’s populations depending on the substrate that they grow on and are not indicative of their age (table S4). This sampling design enabled the collection of individuals from contrasting environments regarding POMS exposure with “nonfarming” and farming areas hosting a high proportion of susceptible and resistant oysters, respectively (Fig. 1A). All individuals were brought to Ifremer facilities in Palavas-les-Flots (Montpellier, France) where they were individually labeled according to previously tested methods (*40*) and acclimatized in 45-liter tanks for 14 days. In each tank, the seawater temperature was increased by 1°C/day from the sampling site temperature to 21°C, continuously ultraviolet C–filtered (BIO-UV), and renewed (30%/hour). During the acclimatization period, all populations were kept in isolated tanks and fed ad libitum with Shellfish Diet 1800 Feeds (Reed Mariculture Inc.).

### Experimental infection by cohabitation between donors and recipient oysters

To classify each oyster as resistant or susceptible phenotype, we performed an experimental infection mimicking POMS event. For this purpose, we used a randomized complete block design composed of eight tanks of 45 liters (replicates) that host a similar number of oysters from each population. Tanks were placed in a water bath to maintain the temperature at 21°C (chiller/heater apparatus; AQUAVIE ICE 3000). In each tank, a water pump (1000 liter/hour; Aquarium System, MaxiJet) and air bubbling maintained water motion and O_2_ level at saturation. Salinity was adjusted to 35 g/liter.

To mimic POMS, a cohabitation protocol was used as described. This started with the injection of 200 μl of an equimolar mix (6.0 × 10^7^ genomic units) of OsHV-1 suspension originated from three different oyster production basins (La Tremblade, Rade de Brest and Thau lagoon) into the adductor muscle of pathogen-free donor oysters. The virus was identified as the OsHV-1 μVar in previous studies performed on oysters originating from the same area at the same sampling period (*41*). Donor oysters will develop the disease and transmit it through the natural infectious route to the recipient oysters (B1 to B6 populations; Fig. 1B). The ratio between donor and recipient oysters was 1:1 in each tank. The donor oyster populations were composed of 50% of the POMS-susceptible biparental oyster family (expected susceptibility, >80%) referred to as H12 (*18*) and 50% of a genetically diversified standardized oyster spats (multiparental; expected susceptibility, ~50%) referred to as NSI.

Immediately after the OsHV-1 μVar injection, donors were equally distributed in the eight experimental tanks (replicates), and 24 hours later, the cohabitation started with the addition of the recipient oysters in each tank. Last, each tank hosted the same number of H12 and NSI donor oysters and the same number of recipient oysters from each population (B1 to B6). Disease progression in donor oysters (dead versus alive) was monitored twice a day (dead donor oysters were removed) during the first 192 hpc, after which they were removed from all experimental tanks. Disease progression in recipient oysters started at 24 hpc and was performed every 2 hours for 360 hours (no mortalities occurred after day 14).

An oyster was classified as moribund or recently died when it could not keep close or close completely its valves after 30 s of emersion (movie S1). Oyster collection at a moribund (susceptible) status enabled the sampling of susceptible oysters before death to avoid DNA degradation. Resistant oysters corresponded to the individuals that were still alive at the end of the experiment (when no death was recorded for 48 hours in all eight tanks). This phenotype was further coded either as a binary trait with the values 0 and 1 corresponding to susceptible and resistant individuals, respectively, or as a semiquantitative trait corresponding to the survival time (expressed in hours) of an individual after its exposure to the OsHV-1 μVar (i.e., the whole duration of the experiment for the resistant oysters; data S1). Upon collection, the flesh of susceptible and resistant oysters was immediately snap-frozen in liquid nitrogen and stored at −80°C until DNA extraction.

### Survival analysis

Differences in oysters’ survival among the six populations were investigated by Kaplan Meyer model with the “survfit” and “ggsurvplot” functions of the survival (v3.2-11) and survminer (v0.4.9) packages, respectively (*42*, *43*). The Cox proportional hazard model was performed using the “coxph” function from survival and was plotted by “ggforest” function from the survminer package. Results were considered significant below the 5% error level.

### Viral load quantification (OsHV-1 μVar)

During the first 192 hpc, 1 ml of water from each experimental tank was sampled daily for viral load quantification. The OsHV-1 μVar DNA was extracted from 200 μl of water using the QIAamp DNA Mini Kit (QIAGEN). Quantitative polymerase chain reaction (qPCR) was performed with 5 μl of DNA using the primers and qPCR parameters previously described (*44*, *45*).

### DNA extraction

Oyster flesh was ground and tissue-homogenized in liquid nitrogen using 50-ml stainless steel bowls and 20-mm-diameter grinding balls with a vibrational frequency of 30 oscillations per second for 30 s (Retsch MM 400 mill). The resulting powder (~20 mg) was used for DNA extraction using the NucleoSpin Tissue Kit following the manufacturer’s instructions (MACHEREY-NAGEL GmbH & Co. KG). DNA quantity and purity were checked with a NanoDrop One spectrophotometer (Thermo Scientific), and quality was checked by 0.8% agarose gel electrophoresis (fig. S6). The extracted DNA was stored at −20°C.

### Exome capture and sequencing

The *C. gigas* exome was captured using the SeqCap Epi Enrichment System protocol (Roche Sequencing Solutions Inc.) (*46*). To capture exons, probes complementary to the whole exonic regions were developed from the genome V9 of *C. gigas* (*47*) in collaboration with Roche. For optimal coverage of the 5′ and 3′ ends of each exon, the probes were designed to cover the 100 base pairs (bp) upstream and downstream to each exon start/end coordinates (data S15).

Exome capture of bisulfite-converted libraries was done according to the manufacturer’s instructions (*46*). Briefly, for each oyster, genomic DNA fragmentation was performed on 1 μg of DNA in addition to DNA phage lambda (GenBank Accession NC_001416) as a spike-in control for the bisulfite conversion efficiency quality control. DNA fragments of an average of 200 bp were obtained by sonication with the Covaris S220 apparatus (Covaris Inc.) using the following parameters (peak incidence power, 175; duty factor, 10; cycle/burst, 200; and duration, 70 s). After end repair and A-tailing, methylated indexed adapters were ligated, and 20 μl of cleaned DNA fragments were subjected to sodium bisulfite conversion using the EZ DNA Methylation-Lightning Kit (Zymo Research, CA). Preamplification of the bisulfite-converted library was carried out for an equimolar pool of eight samples and was then subjected to exome capture at 47°C for 45 hours. After cleaning, a final post-capture PCR amplification was performed and captured bisulfite-converted libraries were sequenced (30×) with an Illumina NextSeq 550 system (PE 2 × 150 bp) or an Illumina NovaSeq S1 6000 system (PE 2 × 100 bp).

### SNP and DNA methylation calling

Raw read quality was checked with FastQC (v0.53) (*48*). Adapter trimming and quality filtering were done with TrimGalore (v0.4.0) (*49*). Bisulfite conversion efficiency was estimated with BSMAPz (v1.1.3) (*50*). BSMAP (v2.90) (*51*) was used to align reads to reference genome V9 of *C. gigas* (*47*). Duplication due to PCR or overlapping between forward and reverse reads was removed following six successive steps (fig. S5): (i) Reads were split into four sets as the top (++ and +−) and the bottom strands (−+ and −−) using the “split” option from BamTools (v1.0.14) (*52*). (ii) The top (++ and +−) and bottom (−+ and −−) strands were merged to produce a set of top and bottom reads using the “merge” function from BamTools. (iii) The top and bottom reads were sorted using the “sort” function from SAMtools (v1.9) (*53*). (iv) The PCR duplicates were removed with “MarkDuplicates” function from Picard (v2.21.1) (*54*). (v) The top and bottom read sets were merged back using the merge option from BamTools. (vi) The overlapping read pairs were clipped using “clipOverlap” function from bamUtil (v1.0.14) (*55*). The scripts are provided (Supplementary Text).

To maximize the accuracy of SNP calling, we used a combination of two SNP callers, FreeBayes (v1.3.1) dedicated to SNP calling from population data and MethylExtract (v1.9) dedicated to SNP calling from bisulfite-converted sequences (*56*). FreeBayes was first used to call the SNPs present in the dataset including those due to the bisulfite conversion (parameters: --use-best-n-alleles = 2, --use-mapping-quality, --no-partial-observations, --min-repeat-entropy = 1). Second, MethylExtract was used to call SNPs that were not due to the bisulfite conversion (C/T SNP; parameters: minQ = 20, minDepthSNV = 8, methNonCpGs = 0.9, maxStrandBias = 0.7, varFraction = 0.1, maxPval = 0.05). Last, only the SNPs identified by both callers were kept and used for GWA study mapping (Supplementary Text).

DNA methylation calling in the CG context (CpGs) was done using MethylExtract (same parameters as mentioned above). The methylation data of all samples were combined and used for EWA mapping (Supplementary Text).

### GWA and EWA quality control

According to the best practices for GWA mapping, the following filtering criteria were applied under the PLINK environment (v1.9): (i) SNPs supported by a coverage of 8× to 150× were kept. (ii) SNPs and samples with a level of missing data above 5% were discarded. (iii) SNPs with a minor allele relative frequency below 0.05 were discarded. (iv) SNPs displaying a significant deviation from Hardy-Weinberg equilibrium (HWE) in resistant (HWE *P* < 1 × 10^−6^) and susceptible oysters (HWE *P* < 1 × 10^−10^) were excluded. (v) Samples with an SD of 3 units from the mean heterozygosity rate of all samples were discarded. (vi) If present, closely related individuals were excluded to remove cryptic relatedness.

For EWA mapping, the following criteria were performed: (i) CpGs supported by a coverage of 8× to 150× were kept. (ii) CpGs and samples with missing data levels above 5% were discarded.

For both datasets, the absence of genetic and epigenetic structure between the six populations was checked using an analysis of multivariate homogeneity of group dispersions with the “betadisper” function of the vegan package (v2.5-7) (*57*). Hierarchical clustering analysis (Euclidian method) and PERMANOVA were performed using the “adonis” function from the vegan package.

### GWA and EWA mapping

GWA mapping was performed by associating SNPs to the binary trait (using a chi-square allelic test with 1 degree of freedom) and the semiquantitative trait (using an asymptotic version of Student’s *t* test) under the PLINK environment (v1.9) (*58*). EWA mapping was performed by associating DNA methylation variation at each CpGs with the binary and semiquantitative traits using a linear regression *t* test (“cpg.assoc” function) from CpGassoc package (v2.60) (*59*). For both GWA and EWA mappings, the significant level of association was defined with an FDR of 0.05. The GWA/EWA mapping results were visualized using quantile-quantile plots and Manhattan plots were generated with qqman package (v0.1.8) (*60*). To benefit from a new reference genome assembly with a chromosomal anchor, homemade scripts were used to position and visualize SNPs and CpGs on chromosomes (Supplementary Text).

### Gene Ontology enrichment analysis

SNPs and CpGs significantly associated with phenotypic variation were subjected to Gene Ontology term enrichment to identify BPs enriched (data S16). This was done using a rank-based Gene Ontology analysis with adaptive clustering following the GO_MWU protocol (*61*). The continuous measure of significance used was −log (*P* value). The following parameters were used for the adaptive clustering: largest = 0.4, smallest = 10, and clusterCutHeight = 0.25. A GO-term was considered enriched when FDR < 0.05. We used the REVIGO tool to visualize significant GO-terms in a semantic similarity relationship obtained from GO_MWU outcome, with the UniProt database and an aggregation value of 0.7 with SimRel as a semantic similarity measure.

### Genetic and epigenetic correlation and association

Correlative (Mantel test) and association MethQTL approaches between genetic and epigenetic variation were adopted to investigate their relationships. On the basis of the Spearman correlation coefficient, Mantel test was applied to estimate the correlation between the genetic and epigenetic matrices of dissimilarity among samples. The association between SNPs and CpG methylation levels was identified using a linear regression implemented in GEM package (v1.24.0) according to the following “Gmodel”: lm(*G* ~ *M* + covariate), where *G* and *M* are the genetic and the DNA methylation level matrix and covariate is the phenotypic trait. This model was applied with the binary and the semiquantitative trait separately.

### Genetic and epigenetic variation partition

Variation partitioning is a method of using the coefficient of determination to fraction the variation of a response variable into four explanatory variables (*62*). Two of them correspond to the fractions of variance exclusively explained by one of the two explanatory matrices (e.g., genetic or epigenetic); one corresponds to the fraction of variance shared by the two explanatory matrices (i.e., genetic and epigenetic), and the last one corresponds to the fraction of the variance unexplained by the model (Supplementary Text).

To estimate the relative contribution of genetic and epigenetic variation to phenotypic variation, we used a method developed by and applied in (*63*). Briefly, the genetic and epigenetic variance was surrogated by producing principal components analyses on the same datasets that were used for GWA/EWA mapping using the “prcomp” function (v2.0.0.) stats package. Then, using a forward selection method implemented in the “ordistep” function in the vegan package (v2.5-7), the best models explaining variance for the binary and semiquantitative traits were separately obtained with genetic and epigenetic principal components (PCs), resulting in four independent models: 2 phenotypic traits × 2 genomic/epigenomic PCs. For each phenotypic trait, the selected PCs from genetic and epigenetic models were retrieved and analyzed in a variation partitioning analysis using the “varpart” function from the vegan package (v2.5-7).

## Supplementary Materials

### This PDF file includes:

Tables S1 to S4 Figs. S1 to S7 Supplementary Text Legend for movie S1 Legends for data S1 to S16
