# Supplementary material for: Epigenetic variations are more substantial than genetic variations in rapid adaptation of oyster to Pacific oyster mortality syndrome
Source: Sci Adv. 2023 Sep 8;9(36):eadh8990. doi: 10.1126/sciadv.adh8990 (PMC10491289; doi:10.1126/sciadv.adh8990)
Supplement: Supplementary file 1 — Tables S1 to S4 Figs. S1 to S7 Supplementary Text Legend for movie S1 Legends for data S1 to S16 [file sciadv.adh8990_sm.pdf]

Science Advances
AAAS

Supplementary Materials for

Epigenetic variations are more substantial than genetic variations in rapid adaptation of oyster to Pacific oyster mortality syndrome

Janan Gawra *et al.*

Corresponding author: Jeremie Vidal-Dupiol, jeremie.vidal.dupiol@ifremer.fr

Sci. Adv. **9**, eadh8990 (2023)
DOI: 10.1126/sciadv.adh8990

The PDF file includes:

Tables S1 to S4
Figs. S1 to S7
Supplementary Text
Legend for movie S1
Legends for data S1 to S16

Other Supplementary Material for this manuscript includes the following:

Movie S1
Data S1 to S16

Supplementary Tables

Table S1. Localization and number of oysters sampled in each population

Area	Population	Latitude	Longitude	Number of oysters
Non-farming	B1	48.379364	-4.446286	61
	B2	48.341789	-4.441086	59
	B3	48.322392	-4.454078	61
	B4	48.296575	-4.451778	56
Farming	B5	48.32815	-4.321947	59
	B6	48.34695	-4.338986	60
			Total	356

Table S2. Hazard ratios of the relative risk of mortality for all six oysters populations.

Area	Population	Number of oysters	Hazard ratio (95%, CI)	*P*-value
Non-Farming	B1	61	Reference *	NA
	B2	59	0.595 (0.3732-0.95)	*0.029*
	B3	61	10550 (0.6840-1.61)	0.825
	B4	56	0.767 (0.4831-1.22)	0.261
Farming	B5	59	0.046 (0.0143-0.15)	*< 0.001*
	B6	60	0.030 (0.0072-0.12)	*< 0.001*

* B1 is the reference

#Events: 150; Global *P*-value (Log-Rank): $2.2141e^{-27}$

AIC: 1563.55; Concordance index: 0.74

Table S3. A large fraction of the epuigenetic variation is associated to the genetic variation

Methylation Quantitative Trait Loci (MethQTL) using the binary or semi-quantitative trait associate with Pacific Oyster Mortality Syndrome as covariate (POMS)

MethQTL	Binary	Semi-quantitative
**Total SNPs**	214,263	214,263
**Total CpGs**	635,201	635,201
**Significant SNP-CpG pairs**	5,151,194	5,152,611
**Total non-redundant SNPs**	160,325	160,220
**Total non-redundant CpGs**	557,703	557,850
**MethQTL controlling a CpG identified in EWA mapping**	207	198
**MethQTL identified by the GWA mapping (suggestive threshold)**	78	95
**CpGs identified by the EWA mapping and controlled by a MethQTL**	126	111
**MethQTL involving a CpG and a SNP identified by the EWA and GWA mapping**	17	12

Table S4. Length and width of the oyster populations sampled

		B1	B2	B3	B4	B5	B6
Length mm	Mean	42.2	45.8	38.1	37.3	43.2	51.0
	SD	7.3	7.1	8.1	9.5	5.7	9.2
	Max	59.5	62.5	55.2	59.4	56.0	75.0
	Min	29.5	34.7	4.5	21.2	32.0	27.0
Width mm	Mean	44.3	44.9	34.8	13.2	29.8	31.4
	SD	8.4	7.2	7.4	6.5	5.4	6.0
	Max	62.0	62.4	50.0	36.0	45.0	43.0
	Min	28.8	31.4	8.0	3.1	20.0	19.0

Supplementary Figures

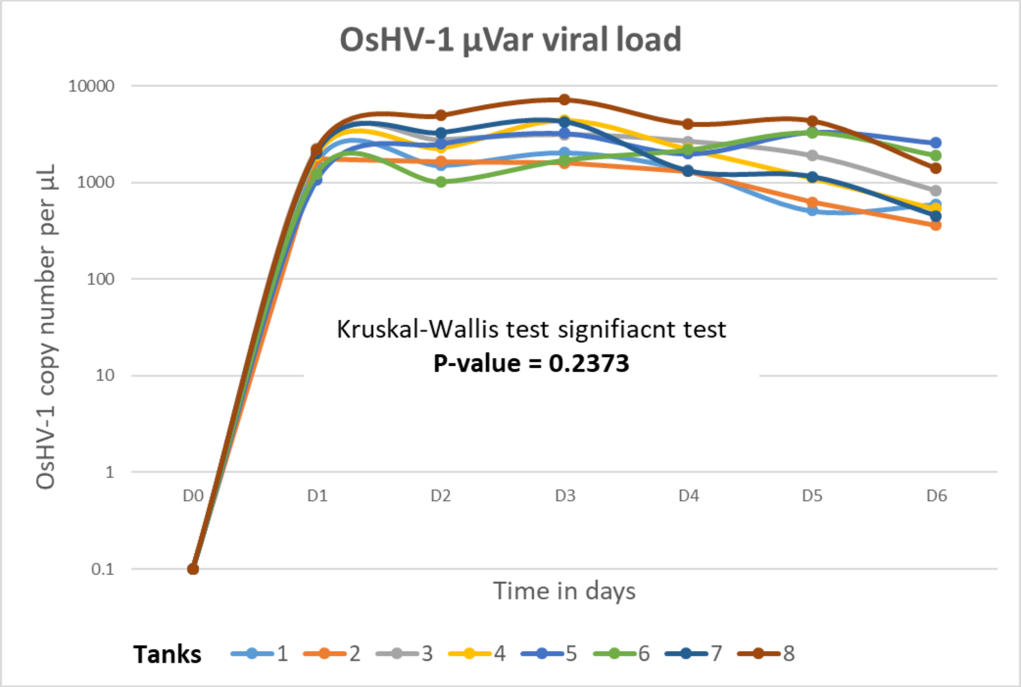

Figure S1. OsHV-1 µVar load in seawater picked 24 hours post donor oyster injection. Quantification of OsHV-1 µVar load in seawater during the first seven days post injection. No significant differences were detected (Kruskal-Wallis test, *P* = 0.24)

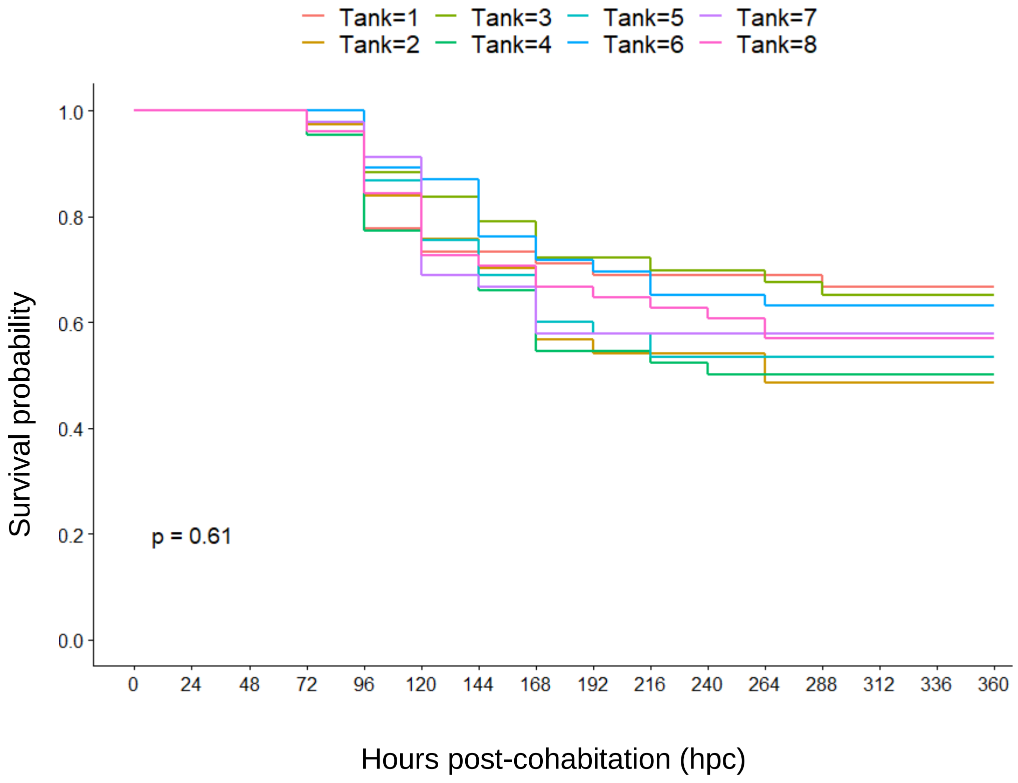

Figure S2. No significant differences of mortality were detected between tanks. The Kaplan–Meier survival curves of oysters among the eight experimental tanks and Log-Rank test do not revealed significant different mortalities between tanks (*P* = 0.61).

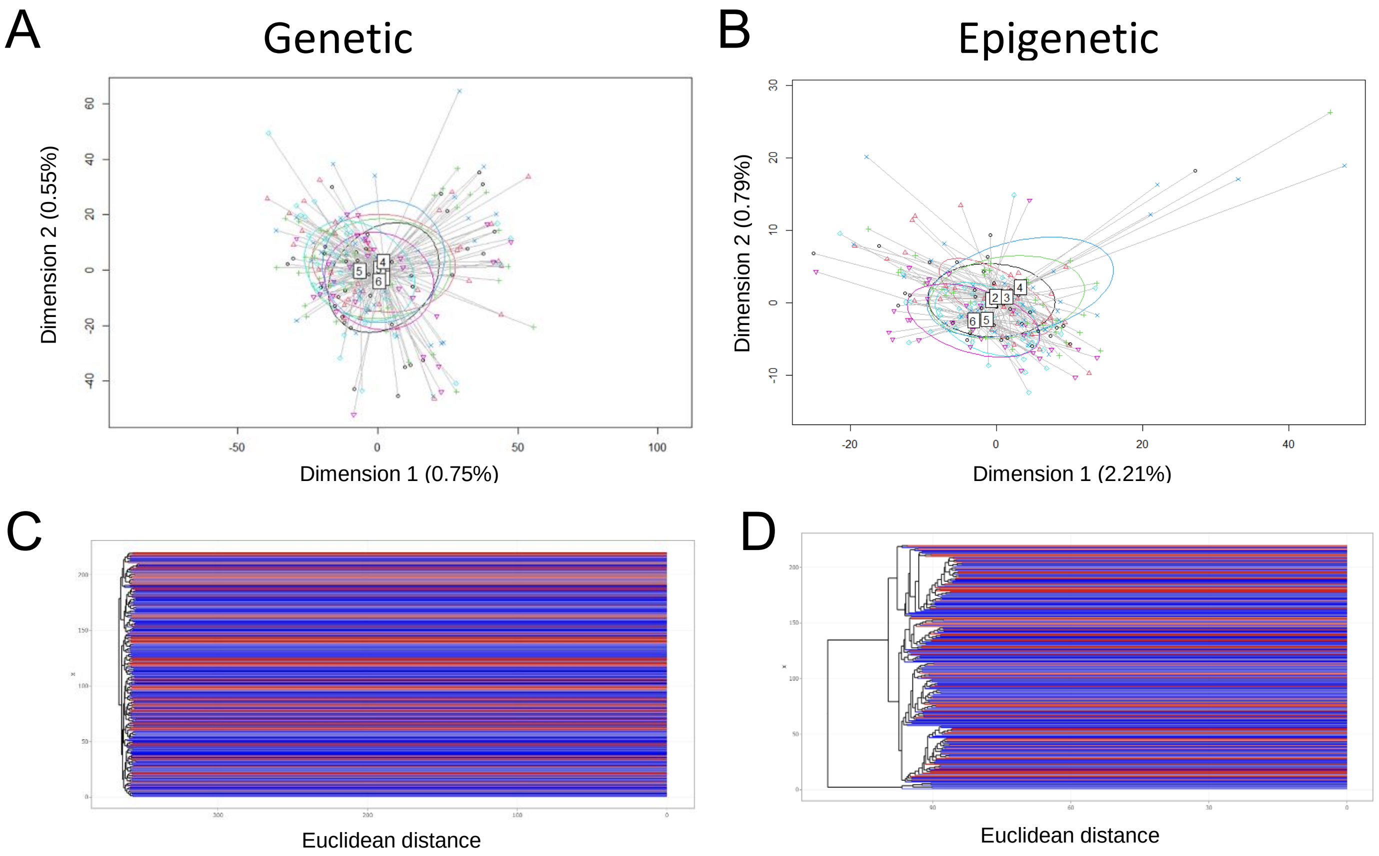

Figure S3. Variance and clustering analysis of the six populations do not revealed population structure

Multivariate homogeneity of group dispersions (variances) **A)** Genetic and **B)** Epigenetic data. Hierarchical cluster analysis for the **C)** Genetic and **D)** Epigenetic data. The blue and red color corresponds to the “Non-farming” and “Farming” populations respectively.

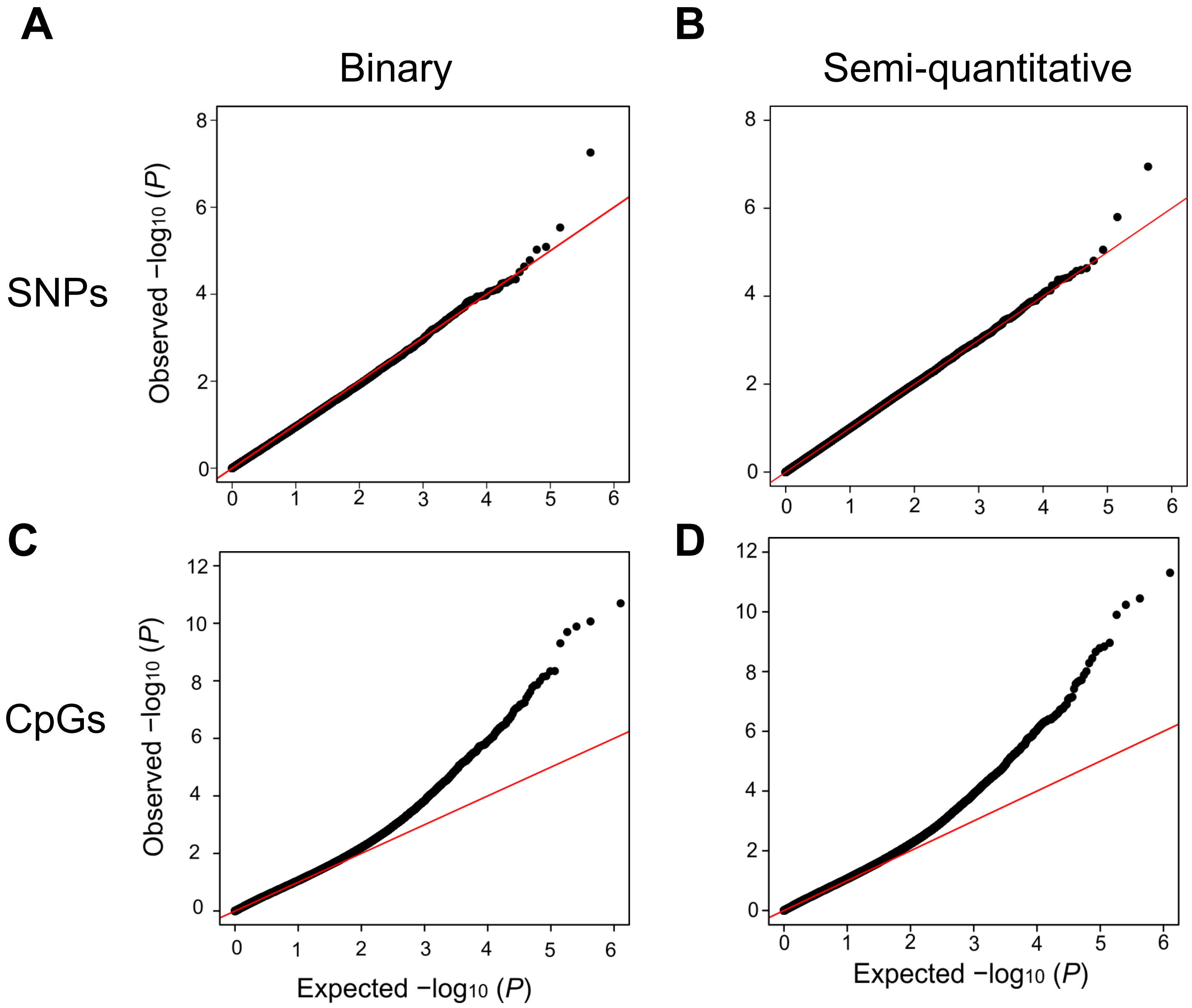

Figure S4. Q-Q plot obtained from the GWA and EWA mapping

The quantile-quantile (Q-Q plot) of the significant SNPs identified in A) binary and B) semi-quantitative trait, and Q-Q plot of the significant CpGs in C) binary and D) semi-quantitative trait.

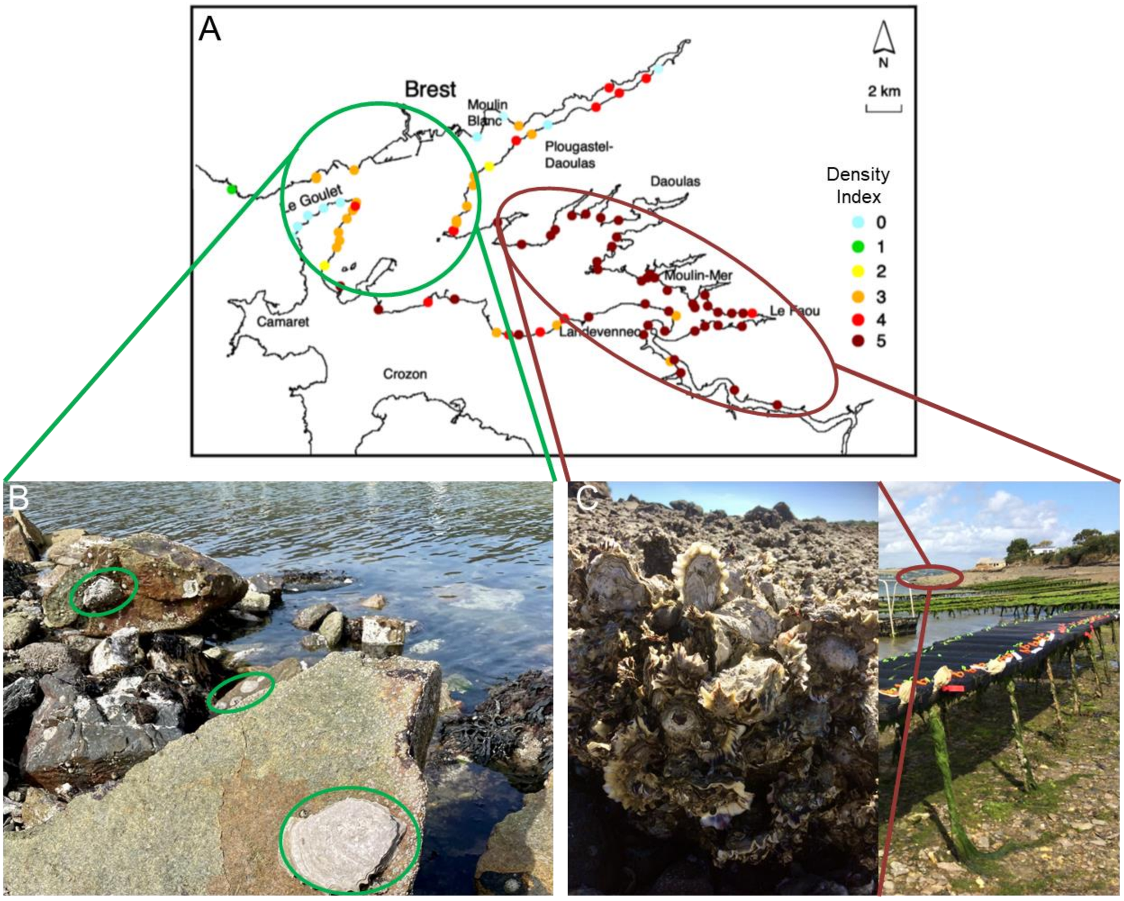

Figure S5. Density of wild oyster populations are lower in non-farming area than in farming area.

A) Distribution of wild oyster density in 2006 in the Rade de Brest, non-farming area (green circle), farming area (red circle). Adapted from Lejart (7), **B)** Photo from Dellec showing a non-farming area characterized by a low oyster density population (density index: from 1 to 3). **C)** Photo of Lagona Daoulas showing a farming area where natural oyster beds of hundreds of wild oysters (density index: 5) co-colocalize with oyster farms.

Density index: 0 no individuals (ind.) m²; 1 < 0.01 ind. m²; 2 between 0.01 and 1 ind. m²; 3 between 1 and 10 ind. m²; 4 between 10 and 100 ind.m²; and 5 between 100 and 1000 ind. m².

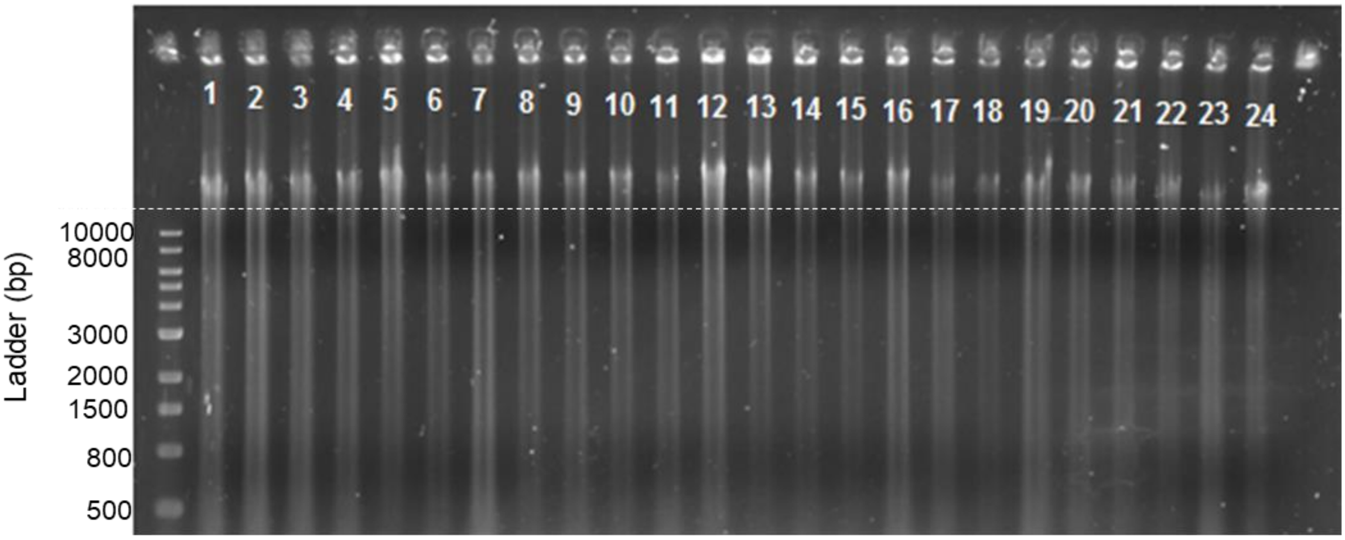

Figure S6. A major band of DNA above 10kb illustrate the suitability of the DNA for exome capture.

Agarose gel electrophoresis (0.8%) performed to evaluate the integrity of genomic DNA (gDNA) extracted from 24 oyster samples (1-12 resistant; 13-24 suceptible). The main criterion for considering a sample suitable for further analysis is the presence of a main gDNA band above the 10,000 base pairs (bp) marker on the ladder track.

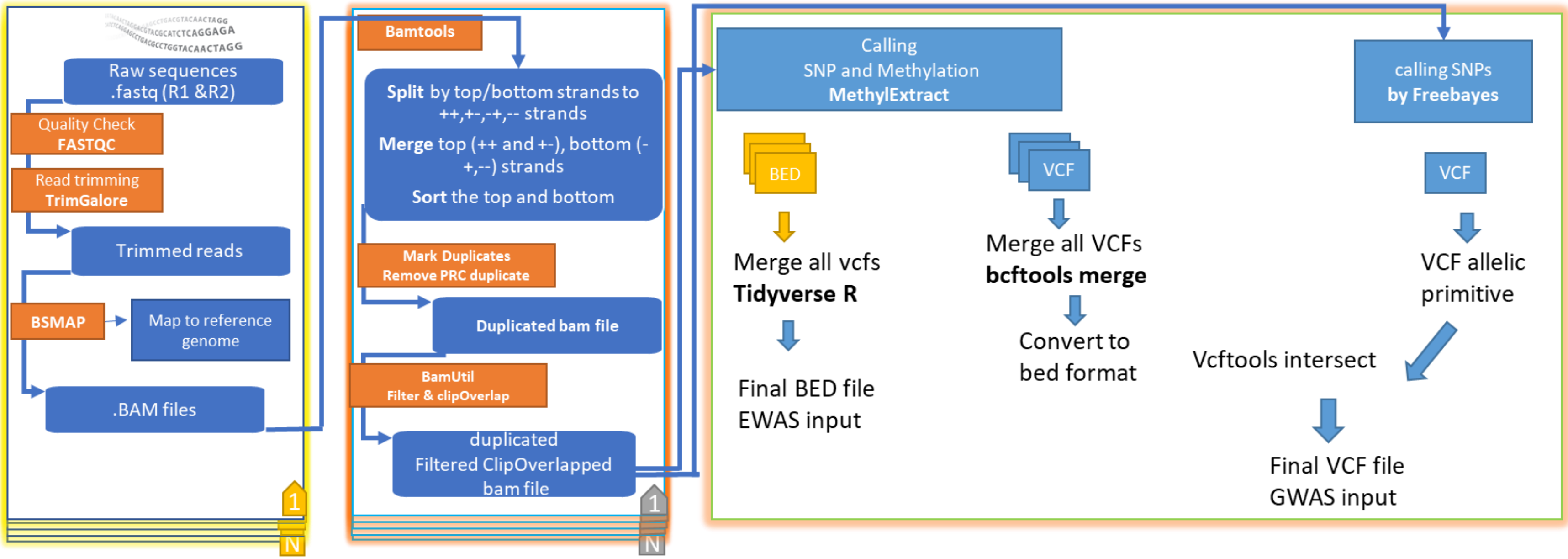

Figure S7. Bioinformatics pipeline applied for the identification of SNP and DNA methylation callings.

Supplementary Information

Bioinformatic scripts used for the different step of our analysis

SNP and DNA methylation Calling

Before the first step - trimming the data to remove the adapter and quality check.
The parameters for trimming: For 150 bp, 2x Paired End reads [remove the last 50]

```
trim_galore --paired --illumina --quality ${params.quality} --three_prime_clip_R1 50  --three_prime_clip_R2 50
```

While for trimming reads with 100 bp 2x Paired End reads

```
trim_galore --paired --illumina --quality ${params.quality} --clip_R1 1  --clip_R2 1
```

Then to do the Mapping - filtering and SNP - Methylation calling
The pipeline for this is ready. On gitlab Ifremer
https://gitlab.ifremer.fr/bioinfo/nf-core-gem.git

Note is better to select only samples that have a closely number of reads otherwise it will lead to many missing data in the VCF (SNP) and Bed (DNA methylation) files

Step 1 - Map reads to *reference* genome using bsmap

```
bsmap -r ${params.bsmap_repeat} -n ${params.bsmap_mapstrand} -s ${params.bsmap_seedsize} -p ${task.cpus} -d ${params.genome} -a ${name}_R1_val_1.fq -b ${name}_R2_val_2.fq -o ${name}.sam &> bsmap-${name}.log 2>&1

picard -Xms512m -Xmx${task.memory.toGiga()}g  AddOrReplaceReadGroups RGID=${name} RGLB=${name} RGPL=illumina RGSM=${name} RGPU=@A00902:117:HKKNJDRXX:2 VALIDATION_STRINGENCY=LENIENT  I=${name}.sam O=${name}.bam &> picard-${name}.log 2>&1
```

STEP 2 - Split, merge and sort mapped reads using bamtools

```
bamtools split -tag ${params.bamtools_tag} -in ${bam} &> bamtools-${name}.log 2>&1
   bamtools merge -in ${name}.TAG_${params.bamtools_tag}_++.bam -in ${name}.TAG_${params.bamtools_tag}_+-.bam -out ${name}_top_merged.bam &>> bamtools-${name}.log 2>&1
   bamtools merge -in ${name}.TAG_${params.bamtools_tag}_-+.bam -in ${name}.TAG_${params.bamtools_tag}_--.bam -out ${name}_bottom_merged.bam &>> bamtools-${name}.log 2>&1
   samtools sort ${name}_top_merged.bam > ${name}_top_merged_sorted.bam 2> samtools-${name}.log
   samtools sort ${name}_bottom_merged.bam > ${name}_bottom_merged_sorted.bam 2>> samtools-${name}.log
```

STEP 3 - Mark duplicates with picard tools - remove duplicates

```
picard -Xms512m -Xmx${task.memory.toGiga()}g -Djava.io.tmpdir=./picard MarkDuplicates \
   VALIDATION_STRINGENCY=SILENT \
   INPUT=${topbam} \
   OUTPUT=${name}_top_rm_dupl.bam \
```

```
    METRICS_FILE=${name}_top_rm_dupl_metrics.txt \
    ASSUME_SORTED=TRUE \
    REMOVE_DUPLICATES=TRUE \
    CREATE_INDEX=TRUE &> ${name}_top_picard.log 2>&1

    picard -Xms512m -Xmx${task.memory.toGiga()}g -Djava.io.tmpdir=./picard MarkDuplicates \
    VALIDATION_STRINGENCY=SILENT \
    INPUT=${bottombam} \
    OUTPUT=${name}_bottom_rm_dupl.bam \
    METRICS_FILE=${name}_bottom_rm_dupl_metrics.txt \
    ASSUME_SORTED=TRUE \
    REMOVE_DUPLICATES=TRUE \
    CREATE_INDEX=TRUE &> ${name}_bottom_picard.log 2>&1
```

STEP 4 - Merge reads with bamtools

```
bamtools merge -in ${topbam_rm_dupl} -in ${bottombam_rm_dupl} -out
${name}_bsmap_non_masked_rm-dupl.bam &> bamtools-${name}.log 2>&1
```

STEP 5 - Filter merged reads with bamtools

```
bamtools filter \
      -isMapped true \
      -isPaired true \
      -isProperPair true \
      -forceCompression \
      -in ${merged_bam} \
      -out ${name}_filtered.bam &> bamtools-${name}.log 2>&1
```

STEP 6 - Filter clipoverlap with bamutils and index bam file

```
bam clipOverlap \
   --stats \
   --in ${filtered_bam} \
   --out ${name}_clipped.bam &> bamtutils-${name}.log 2>&1
```

STEP 7 - Methylation maps and SNP calling with MethylExtract

```
MethylExtract.pl p=${task.cpus} seq=${params.genome} inDir=. outDir=.
minDepthMeth=${params.methylextract_mindepthmeth}
minDepthSNV=${params.methylextract_mindepthsnv} context=ALL wigOut=Y bedOut=Y
flagW=${params.methylextract_flagw} flagC=${params.methylextract_flagc} &>
${name}_methylextract.log
```

THEN IS MERGE THE BED FILES AND VCF FILES

Two problems here to deal with. That maybe come from the not well having a homogenous number of reads, if a closely number of reads have selected maybe these problem probably won't show up.

First: merge the vcf files (Single Nucleotide Polymorphisms; SNPs containing file)

Because with the METHYLEXTRACT package the SNP calling is done based on single sample producing single vcf file reporting only the SNPs. When merging many VCF files from different sample would lead to many missing data. Simply because one sample or many samples would have a homozygote genotype for reference allele and others would have a heterozygote or homozygote genotype for alternative allele.

How to tackle this issue?
First, we use FreeBayes to obtain a single VCF file for all the samples.
Script for FREEBAYES

First, we use FreeBayes to obtain a single VCF file.

```
#PBS -q omp
#PBS -l walltime=120:00:00
#PBS -l mem=115g
#PBS -l ncpus=56
## Manage script history
INPUT_DIR=/home/datawork-ihpe/gem/06_clipped-bam-files
FreeBayes_TOOLS=". /appli/bioinfo/freebayes/latest/env.sh"
#GENOME=/home1/datawork/jgawra/GWAS_EWAS_TEST/vcfcd $INPUT_DIR
$FreeBayes_TOOLS

#########################################
## Shell variables ##
#########################################
#INPUT_DIR=/home1/datawork/jgawra/GWAS_EWAS_TEST
GENOME=/home1/datawork/jgawra/GWAS_EWAS_TEST/vcf/oyster.v9.fa
OUTPUT_DIR=/home1/scratch/jgawra/GWAS_EWAS_TEST

#########################################
## prepapre input file ##
#########################################

 ls -d "${INPUT_DIR}/"*"_clipped.bam" > "${INPUT_DIR}/SAMPLES_clipped_bam.txt"

#########################################
## Freebays variant calliing ##
#########################################
echo "create a list of bam files... "`cat "${INPUT_DIR}/SAMPLES_clipped_bam.txt"`
echo "variant calling..."
freebayes-parallel <(fasta_generate_regions.py "${GENOME}.fai" 10000) 56 -p 2 -f $GENOME --use-
best-n-alleles 2 --use-mapping-quality --min-coverage 8 --no-partial-observations --min-repeat-
entropy 1 -L ${INPUT_DIR}/SAMPLES_clipped_bam.txt >&
${OUTPUT_DIR}/freebayes_248_Brest_samples_s
econd_try.vcf 2> ${OUTPUT_DIR}/freebayes_248_Brest_samples_second_try.vcf.log
```

Then I need to check if the vcf file is ok. Then is to change the vcf from the haplotype to single SNP type by using the below script

```
#!/usr/bin/env bash
#PBS -q omp
#PBS -l walltime=250:00:00
#PBS -l mem=115g
#PBS -l ncpus=56
DATA=/home/datawork-ihpe-gem-nos
BCFTOOLS_TOOLS=". /appli/bioinfo/vcflib/1.0.0_rc1/env.sh"
cd $DATA
$BCFTOOLS_TOOLS
```

```
vcfallelicprimitives -kg  freebayes_248_Brest_samples_second_try_remove-bad-lines.vcf  > freebayes_248_Brest_samples_second_try_remove_bad_lines_vcfallelicprimitives.vcf
```

FreeBayes is not able to differentiate a real SNP fro independently in separate VCF files for each sample re, MethylExtract was used to call real SNPs in separate VCF files for each sample independently. This is done in STEP 7 already [page 4].

Then, we used BCFTOOLS to merge all the VCF files into a single VCF file and convert it to a bed file format (which contains the real SNP genomic location).

```
#!/usr/bin/env bash
#PBS -q sequentiel
#PBS -l walltime=00:30:00
#PBS -l mem=5g
DATA=/home1/datawork/jgawra/GWAS_EWAS_TEST/vcf/vcf-sub
BCFTOOLS_TOOLS=". /appli/bioinfo/bcftools/latest/env.sh"
cd $DATA
$BCFTOOLS_TOOLS

#### First is to bgzip the vcf file to index it.
for file in *_sub.vcf
do
	bgzip -c $file
done
#### Then to index it.
#for file in *.vcf.gz ; do bcftools index -c $file ; done
#### Finally to merge it.
#bcftools merge --force-samples *vcf.gz -Oz -o Merged.vcf.gz
```

Then is to make a bed file like to of all the vcf position (the real SNPs) that to be used in the next step

Finally, we intersected it with a VCF file produced by FreeBayes using vcfintersect from VCFTOOLS (version 0.1.16) and obtained the final VCF file that was will be used for GWA mapping analyses.

```
#!/usr/bin/env bash
#PBS -q omp
#PBS -l walltime=50:00:00
#PBS -l mem=50g
#PBS -l ncpus=28

DATA=/home/datawork-ihpe-gem-nos
BED_DATA=/home/datawork-ihpe/gem/06_clipped-bam-files
#BCFTOOLS_TOOLS=". /appli/bioinfo/vcflib/1.0.0_rc1/env.sh"
VCFTOOLS=". /appli/bioinfo/vcftools/latest/env.sh"
cd $DATA
$VCFTOOLS

Vcftools
--vcf freebayes_248_Brest_samples_second_try_remove_bad_lines_vcfallelicprimitives.vcf
 --bed ${BED_DATA}/vcf2bed_jb.bed
 --out freebayes_248_samples_vcfallelicprimitive_with_region_methylextract.vcf
  --temp $SCRATCH –recode
#### This file is ready for GWA mapping analysis
####### This is a vcf file that would be used for GWA mapping analysis.
```

Second: How to deal with Bed file

First I used R to merge all the bed files (produced by methyextract in step 7; page 3).
#first I need to prepare the bed files

```
#!/usr/bin/env bash
#PBS -q omp
#PBS -l walltime=48:00:00
#PBS -l select=1:ncpus=28:mem=115g
DATA=/home/datawork-ihpe/gem/09_methylextract_results/
cd $DATA
for i in *.bed
do
 sed '1d' "$i" > ${i%.bed}_temp1.bed
done
for i in *_temp1.bed
do
 awk '{print $1,($3 - 1),($5/10)}' "$i" > ${i}_temp2.bed
done

for i in *_temp2.bed
do
 sed  -i '1i chrom pos  methratio' "$i"
done

for i in *_temp2.bed
do
 sed -e 's/  */\t/g' "$i" > ${i%CG_temp1.bed_temp2.bed}CG_2.bed
done
```

Then is to merge all the bed files

```
Merge <- tibble(chrom = "C13972",
                pos = 83,
                methratio_ValueUseless = 0)

for (file in list.files(path=".", pattern="*CG_2.bed")     ) {
  filename <- file
  Merge <- full_join(Merge,read_tsv(file) %>%
                 rename(!!filename := methratio) %>%
                 as_tibble() )
}
Merged3.bed <- Merge %>% select(-methratio_ValueUseless)
```

This file is ready for EWA mapping analysis

Locating the SNPs and CpGs in the new released Genome of Crassostrea gigas.

SNPs locating in the new genome

Locating the SNPs passing the PLINK quality control to the NEW Roslin GENOME (with chromosome information). This is for visualization purpose, so we can have a Manhattan plot with the ten chromosome.

Preparing a FASTA file

The final plink output file (binary fileset; that was used for GWA mapping; containing 214,263 SNPs) were mapped to the new genome CGA using the vcfprimer from

First the final plink binary fileset were converted to vcf file.

Then for each SNP, a 100 bps were added and finally producing a fasta file that each line is a SNP with 100 bps following.

Script for making a FASTA file by vcfprimer v1.0.0. on Linux.

```
#!/usr/bin/env bash
#PBS -q omp
#PBS -l walltime=40:00:00
#PBS -l mem=20g
#PBS -l ncpus=8
##call the vcflib tool
. /appli/bioinfo/vcflib/1.0.0_rc1/env.sh
## data location
DATA=/home/datawork-ihpe-gem-nos/CGA-genome
cd $DATA
vcfprimers /home/datawork-ihpe-gem-nos/filtered_Depth_vcf_8-150/plink.vcf -f oyster.v9.fa -l 100 >
reads-100bp_plink_214k.fasta
```

Align the fasta file to new genome

Then the fasta file is used to align it on the new genome using the BOWTIE2 v2.3.5

B.1- First we make an index for the genome

```
#!/usr/bin/env bash
#PBS -q omp
#PBS -l mem=50G
#PBS -l ncpus=28
#PBS -l walltime=10:00:00
BANK_DIR=/home/datawork-ihpe-gem-nos/CGA-genome
BANK_FILE_NAME=/home/datawork-ihpe-gem-nos/CGA-
genome/GCA_902806645.1_cgigas_uk_roslin_v1_genomic.fna

# Le nom de la banque tel qu'on l'utilisera avec Bowtie2
INDEX_NAME=/home/datawork-ihpe-gem-nos/CGA-
genome/GCA_902806645.1_cgigas_uk_roslin_v1_genomic

# Lancement de Bowtie
bowtie2_cpus=$((${NCPUS}-2))
. /appli/bioinfo/bowtie2/2.3.5/env.sh

cd ${BANK_DIR}
bowtie2-build  ${BANK_FILE_NAME} ${INDEX_NAME}  -p ${bowtie2_cpus} >&
$BANK_DIR/mkbowtie.log 2>&1
```

B.2- Align the fasta to the new genome and produce a SAM file

```
#!/usr/bin/env bash
#PBS -q omp
#PBS -l mem=50G
#PBS -l ncpus=28
#PBS -l walltime=10:00:00

BANK_DIR=/home/datawork-ihpe-gem-nos/CGA-genome
#BANK_FILE_NAME=/home/datawork-ihpe-gem-nos/CGA-
genome/GCA_902806645.1_cgigas_uk_roslin_v1_genomic.fna

# Le nom de la banque tel qu'on l'utilisera avec Bowtie2
INDEX_NAME=/home/datawork-ihpe-gem-nos/CGA-
genome/GCA_902806645.1_cgigas_uk_roslin_v1_genomic

# launching the BOWTIE2
bowtie2_cpus=$((${NCPUS}-2))
. /appli/bioinfo/bowtie2/2.3.5/env.sh

cd ${BANK_DIR}
bowtie2 -x ${INDEX_NAME} -f ${BANK_DIR}/reads-100bp_plink_414k.fasta  -S reads-
50bp_plink_214.fasta.sam
```

Convert the SAM file to Bed file
Then convert the SAM file were converted to bed using Linux cat, grep and sed and awk tools

```
cat  reads-100bp_plink_214.fasta.sam | grep -v "@HD"  | grep -v "@SQ" |grep -v "@PG"  | awk
'{print $3"\t"$1"\t"($4+100)"\t"$5}'  | sed 's/_LEFT//g' > LR_reads-100bp_plink_214K.fasta.sam.bed
```

The output would be a SNP, and the position of the SNP in the chromosome and its coordinates.
Intersecting with the GWA mapping output file to be used for Manhattan plot

Then this bed file were intersected with the GWA mapping output using the tidyverse package by left_join function in R.

```
library(tidyverse)
setwd("E:/GEM/PhD-Thesis/Lab Methodology/GWAS_EWAS_ANALYSIS/plink/Final_GWAS")
### read the file with the SNPs and their coordinates in the new genome
df <- read.delim("E:/GEM/PhD-Thesis/Lab
Methodology/GWAS_EWAS_ANALYSIS/plink/Final_GWAS/LR_reads-
100bp_plink_214K.fasta.sam.bed", header=FALSE)
## load the GWAS association file to intersect it with the bed file to located each SNP in the new
genome
assoc_results <- read.csv("E:/GEM/PhD-Thesis/Lab
Methodology/GWAS_EWAS_ANALYSIS/plink/Final_GWAS/assoc_results.assoc", sep="")
assoc_results_pq.qassoc <- read.csv("E:/GEM/PhD-Thesis/Lab
Methodology/GWAS_EWAS_ANALYSIS/plink/Final_GWAS/assoc_results-pq.qassoc", sep="")

colnames(df)[1] <- "CHR"
colnames(df)[2] <- "SNP"
colnames(df)[3] <- "POS"
colnames(df)[4] <- "Q"
df2<- cbind(df, read.table(text = as.character(df$CHR), sep = '_'))
```

```
df2$SNP <- paste(df2$V2,df2$V3, sep="_")
df3 <- df2[c(5,1,2,3)]
library(dplyr)
df4 <- df3 %>%
  group_by(SNP) %>%
  filter(POS==max(POS))
df5 =df4[!duplicated(df4$SNP), ]

logistic_adjusted_merged <- left_join(df5, assoc_results , by=c("SNP"))
logistic_adjusted_merged <- left_join(df5, assoc_results_pq.qassoc , by=c("SNP"))

colnames(logistic_adjusted_merged)[1] <- "CHR"

logistic_adjusted_merged$CHR [grepl("CADCXH*", logistic_adjusted_merged$CHR)] <- "88"
logistic_adjusted_merged$CHR [grepl("LR761634.1", logistic_adjusted_merged$CHR)] <-"1"
logistic_adjusted_merged$CHR [grepl("LR761635.1", logistic_adjusted_merged$CHR)] <-"2"
logistic_adjusted_merged$CHR [grepl("LR761636.1", logistic_adjusted_merged$CHR)] <-"3"
logistic_adjusted_merged$CHR [grepl("LR761637.1", logistic_adjusted_merged$CHR)] <-"4"
logistic_adjusted_merged$CHR [grepl("LR761638.1", logistic_adjusted_merged$CHR)] <-"5"
logistic_adjusted_merged$CHR [grepl("LR761639.1", logistic_adjusted_merged$CHR)] <-"6"
logistic_adjusted_merged$CHR [grepl("LR761640.1", logistic_adjusted_merged$CHR)] <-"7"
logistic_adjusted_merged$CHR [grepl("LR761641.1", logistic_adjusted_merged$CHR)] <-"8"
logistic_adjusted_merged$CHR [grepl("LR761642.1", logistic_adjusted_merged$CHR)] <-"9"
logistic_adjusted_merged$CHR [grepl("LR761643.1", logistic_adjusted_merged$CHR)] <-"10"
##
logistic_adjusted_merged$CHR <- gsub("\\*", "99", logistic_adjusted_merged$CHR)
###write.table(logistic_adjusted_merged, file = "ADD_logistic_merged.txt", sep = "\t", quote = FALSE,
row.names =F)
logistic_adjusted_merged$CHR<-as.numeric(logistic_adjusted_merged$CHR)
logistic_adjusted_merged$POS<-as.numeric(logistic_adjusted_merged$POS)
logistic_adjusted_merged$P<-as.numeric(logistic_adjusted_merged$P)
colnames(logistic_adjusted_merged)[2] <- "chr_position_dir"
colnames(logistic_adjusted_merged)[5] <- "chr_v9"
write.table(logistic_adjusted_merged, file ="assoc_results_merged.txt", sep = "\t", row.names =
FALSE)
write.table(logistic_adjusted_merged, file ="assoc_results_pq.qassoc_merged.txt", sep = "\t",
row.names = FALSE)
```

Plotting the Manhattan plot and QQplot

```
library(qqman)
### Manhattan plot
jpeg(filename = "2Manhattan_plot_gwas_binary.jpg", width = 1200, height = 550 )
manhattan(x = assoc_results_merged, chr = "CHR", bp = "POS", p = "P",   genomewideline = -
log10(0.05/214318), suggestiveline = -log10(0.0005),   col = c("blue", "red"))
dev.off()
jpeg(filename = "2Manhattan_plot_gwas_coninous.jpg", width = 1200, height = 550 )
manhattan(x = assoc_results_pq.qassoc_merged, chr = "CHR", bp = "POS", p = "P",   genomewideline
= -log10(0.05/214318), suggestiveline = -log10(0.0005),   col = c("blue", "red"))
dev.off()

####qqplot
jpeg(filename = "qq_plot_gwas_binary.jpg")
```

```
qq(assoc_results_merged$P)
dev.off()
jpeg(filename = "qq_plot_gwas_continous.jpg")
qq(assoc_results_pq.qassoc_merged$P)
dev.off()
```

Intersect the CpG to the new Genome

First prepare bed file
First we prepare a bed file. It is a CpG and its start position and end position for a CpG.

```
CpG_List = results_95_220_max [c(1)]
df2<- cbind(CpG_List, read.table(text = as.character(CpG_List$TargetID), sep = "_"))
CpG_List = df2[c(2,3)]
write.table(CpG_List, file ="CpG_List-635K.txt", sep = "\t",row.names = F, quote = F,col.names = F )
```

Prepare a fasta file
To do this, a getfasta function from bedtools was used to get a fasta file.

```
awk '{print $1"\t"$2"\t"($2+49)"\t"(49-$2)}' CpG_List-635K.txt > tmp1
sed 's/-//g' tmp1 > tmp2
awk '{print $1"\t"$4"\t"$3}' tmp2 > tmp3
bedtools getfasta -fi oyster.v9.fa -bed  tmp3 -name > tmp99
```

Convert the Fasta to bed file

```
cat  2getfasta_CpG_filtered_bed.fasta.sam | grep -v "@HD"  | grep -v "@SQ" |grep -v "@PG"  | awk
'{print $3"\t"$1"\t"($4+49)"\t"$5}'  | sed 's/_LEFT//g' > 2getfasta_CpG_filtered_bed.fasta.sam.bed
```

Intersecting the Bed with EWA mapping output

```
get2<- read.table("E:/GEM/PhD-Thesis/Lab
Methodology/GWAS_EWAS_ANALYSIS/cpgassoc/Final_EWAS/2getfasta_CpG_filtered_bed.fasta.sam
.bed", quote="\"", comment.char="")
get = get2 [c(1,2,3)]
colnames(get)[1] <- "CHR"
colnames(get)[2] <- "CHR_Pos_Start_End"
colnames(get)[3] <- "MAPINFO"

get$CHR <- as.character(get$CHR)
get$CHR [grepl("CADCXH*", get$CHR)] <- "88"
get$CHR [grepl("LR761634.1", get$CHR)] <-"1"
get$CHR [grepl("LR761635.1", get$CHR)] <-"2"
get$CHR [grepl("LR761636.1", get$CHR)] <-"3"
get$CHR [grepl("LR761637.1", get$CHR)] <-"4"
get$CHR [grepl("LR761638.1", get$CHR)] <-"5"
get$CHR [grepl("LR761639.1", get$CHR)] <-"6"
get$CHR [grepl("LR761640.1", get$CHR)] <-"7"
get$CHR [grepl("LR761641.1", get$CHR)] <-"8"
get$CHR [grepl("LR761642.1", get$CHR)] <-"9"
get$CHR [grepl("LR761643.1", get$CHR)] <-"10"

get$CHR <- as.numeric(get$CHR)
#newthing
get$CHR_Pos_Start_End <- gsub (":", "-", get$CHR_Pos_Start_End)

df2<- cbind(get, read.table(text = as.character(get$CHR_Pos_Start_End), sep = "-"))
```

```
df2$TargetID = paste(df2$V1, df2$V2+49,sep="_")

# colnames(df2)[4] <- "TargetID"
# colnames(df2)[5] <- "Pos"
#
# df2$TargetID <- gsub (":", "_", df2$TargetID)

library(tidyverse)
colnames(results_95_220_max)[1] <- "TargetID"
colnames(results_cpg_death2)[1] <- "TargetID"

merge_binary <- left_join(results_cpg_death2, df2, by="TargetID")
merge_continous<- left_join(results_95_220_max, df2, by="TargetID")
merge_binary$CHR[is.na(merge_binary$CHR)] <- 99
merge_binary$MAPINFO[is.na(merge_binary$MAPINFO)] <- 100
merge_continous$CHR[is.na(merge_continous$CHR)] <- 99
merge_continous$MAPINFO[is.na(merge_continous$MAPINFO)] <- 100

write.table(merge_binary, file ="merge_binary_Ewas.txt", sep = "\t", row.names = FALSE)
write.table(merge_continous, file ="merge_continous_Ewas.txt", sep = "\t", row.names = FALSE)

merge_continous_Ewas <-
read.delim("C:/GEM_THESE/Final_EWAS/merge_continous_Ewas_CpG_GENE2.txt")
merge_binary_Ewas <-
read.delim("C:/GEM_THESE/Final_EWAS/merge_Binary_Ewas_CpG_GENE2.txt")

colnames(merge_continous_Ewas)[1] <- "SNP"
colnames(merge_binary_Ewas)[1] <- "SNP"
summary(merge_binary_Ewas)

merge_binary_Ewas$P.value[is.na(merge_binary_Ewas$P.value)] <- 1
merge_continous_Ewas$P.value[is.na(merge_continous_Ewas$P.value)] <- 1

library(qqman)
#### plotting by qqman package

jpeg(filename = "Manhattan_plot_ewas_binary.jpg", width = 1200, height = 550 )
manhattan(x = merge_binary_Ewas, chr = "CHR", bp = "MAPINFO", p = "P.value",   genomewideline =
-log10(1.901340e-05),suggestiveline = FALSE,    col = c("blue", "red"))
dev.off()

jpeg(filename = "Manhattan_plot_ewas_continous.jpg", width = 1200, height = 550 )
manhattan(x = merge_continous_Ewas, chr = "CHR", bp = "MAPINFO", p = "P.value",
genomewideline = -log10(2.147985e-05),suggestiveline = FALSE,    col = c("blue", "red"))
dev.off()

jpeg(filename = "qq_plot_ewas_binary.jpg")
qq(merge_binary_Ewas$P.value)
dev.off()
jpeg(filename = "qq_plot_ewas_continous.jpg")
qq(merge_continous_Ewas$P.value)
dev.off()
```

Variation Partition analyses:

```
setwd("C:/GEM_THESE/Final_distangle_EWAS_GWAS/Varpart")
library(vegan)
#
# ###load the genotype file with no NA
# G_df2 <-
read.delim("C:/GEM_THESE/Final_distangle_EWAS_GWAS/Final_Variation_partiton/genotype_220_
varpart_input_no_NA.txt")
#
#
#
# #### load the saved file
# df_E <-
read.delim("C:/GEM_THESE/Final_distangle_EWAS_GWAS/epigenotype_220_varpart_input_no_NA.t
xt")
#
# #### prepare the file for the PCA
# data <- df_E
# rnames <- data[[1]]# assign labels in column 1 to "rnames"
# mat_data <- data.matrix(data[,2:221])  # transform column 2 - end into a matrix
# rownames(mat_data) <- rnames
# data <- as.matrix(mat_data)
# data2 <- data/(100)
# EE <- data2
#
# ###transpnse the dataframe
# EEE <- t(EE)
# m_E <- data.frame (EEE)
#
#
# df2_G <- G_df2[, names(df_E)]
#
# data <- df2_G
# rnames <- data[[1]]# assign labels in column 1 to "rnames"
# mat_data <- data.matrix(data[,2:221])  # transform column 2 - end into a matrix
# rownames(mat_data) <- rnames
# data <- as.matrix(mat_data)
# GG <- data
# GGG <- t(GG)
# m_G_1 <- data.frame (GGG)


### make the PCA
# meth.pc=prcomp(m_E)
# save(meth.pc, file="meth.pc.Rdata")
load("meth.pc.Rdata")
summary(meth.pc)
# meth.bs=meth.pc$x[,1:220]

# write.table(meth.bs, file ="prcomp_epigenotype_220_varpart_input.txt", sep = "\t",row.names = T)
```

```
#
meth.bs <-
read.delim("C:/GEM_THESE/Final_distangle_EWAS_GWAS/Varpart/prcomp_epigenotype_220_varpa
rt_input.txt")
#
#
# genet.pc=prcomp(m_G_1)
# save(genet.pc, file="genet.pc.Rdata")
load("genet.pc.Rdata")
summary(genet.pc)
# genet.bs <- genet.pc$x[,1:220]
#
# write.table(genet.bs, file ="prcomp_genotype_220_varpart_input.txt", sep = "\t", row.names = T)

genet.bs <-
read.delim("C:/GEM_THESE/Final_distangle_EWAS_GWAS/Varpart/prcomp_genotype_220_varpart_
input.txt")



# Final_phenotype_binary_220 <-
read.delim("C:/GEM_THESE/Final_distangle_EWAS_GWAS/Varpart/Final_phenotype_binary_220.txt
")
#
# df_PP <- Final_phenotype_binary_220[, names(df_E)]
#
#
# data <- df_PP
# rnames <- data[[1]]# assign labels in column 1 to "rnames"
# mat_data <- data.matrix(data[,2:221])  # transform column 2 - end into a matrix
# rownames(mat_data) <- rnames
# data <- as.matrix(mat_data)
# PP <- data
# PPP <- t(PP)
# m_P <- data.frame (PPP)
#
# write.table(m_P, file ="phenotype_bin_220_varpart_input.txt", sep = "\t", row.names = T)

m_P <-
read.delim("C:/GEM_THESE/Final_distangle_EWAS_GWAS/Varpart/phenotype_bin_220_varpart_inp
ut.txt")

# Final_phenotype_continous_220 <-
read.delim("C:/GEM_THESE/Final_distangle_EWAS_GWAS/Varpart/Final_phenotype_continous_220.
txt")
# df_PP_con <- Final_phenotype_continous_220[, names(df_E)]
#
#
#
# data <- df_PP_con
# rnames <- data[[1]]# assign labels in column 1 to "rnames"
# mat_data <- data.matrix(data[,2:221])  # transform column 2 - end into a matrix
```

```
# rownames(mat_data) <- rnames
# data <- as.matrix(mat_data)
# PP <- data
# PPP <- t(PP)
# m_P_con <- data.frame (PPP)
#
# write.table(m_P_con, file ="phenotype_con_220_varpart_input.txt", sep = "\t", row.names = T)

m_P_con <-
read.delim("C:/GEM_THESE/Final_distangle_EWAS_GWAS/Varpart/phenotype_con_220_varpart_in
put.txt")



mod0=rda(m_P~1)

mod1=rda(m_P~genet.bs[, 1]+genet.bs[, 2]+genet.bs[, 3]+genet.bs[, 4]+genet.bs[, 5]+genet.bs[,
6]+genet.bs[, 7]+genet.bs[, 8]+genet.bs[, 9]+genet.bs[, 10]+genet.bs[, 11]+genet.bs[, 12]+genet.bs[,
13]+genet.bs[, 14]+genet.bs[, 15]+genet.bs[, 16]+genet.bs[, 17]+genet.bs[, 18]+genet.bs[,
19]+genet.bs[, 20]+genet.bs[, 21]+genet.bs[, 22]+genet.bs[, 23]+genet.bs[, 24]+genet.bs[,
25]+genet.bs[, 26]+genet.bs[, 27]+genet.bs[, 28]+genet.bs[, 29]+genet.bs[, 30]+genet.bs[,
31]+genet.bs[, 32]+genet.bs[, 33]+genet.bs[, 34]+genet.bs[, 35]+genet.bs[, 36]+genet.bs[,
37]+genet.bs[, 38]+genet.bs[, 39]+genet.bs[, 40]+genet.bs[, 41]+genet.bs[, 42]+genet.bs[,
43]+genet.bs[, 44]+genet.bs[, 45]+genet.bs[, 46]+genet.bs[, 47]+genet.bs[, 48]+genet.bs[,
49]+genet.bs[, 50]+genet.bs[, 51]+genet.bs[, 52]+genet.bs[, 53]+genet.bs[, 54]+genet.bs[,
55]+genet.bs[, 56]+genet.bs[, 57]+genet.bs[, 58]+genet.bs[, 59]+genet.bs[, 60]+genet.bs[,
61]+genet.bs[, 62]+genet.bs[, 63]+genet.bs[, 64]+genet.bs[, 65]+genet.bs[, 66]+genet.bs[,
67]+genet.bs[, 68]+genet.bs[, 69]+genet.bs[, 70]+genet.bs[, 71]+genet.bs[, 72]+genet.bs[,
73]+genet.bs[, 74]+genet.bs[, 75]+genet.bs[, 76]+genet.bs[, 77]+genet.bs[, 78]+genet.bs[,
79]+genet.bs[, 80]+genet.bs[, 81]+genet.bs[, 82]+genet.bs[, 83]+genet.bs[, 84]+genet.bs[,
85]+genet.bs[, 86]+genet.bs[, 87]+genet.bs[, 88]+genet.bs[, 89]+genet.bs[, 90]+genet.bs[,
91]+genet.bs[, 92]+genet.bs[, 93]+genet.bs[, 94]+genet.bs[, 95]+genet.bs[, 96]+genet.bs[,
97]+genet.bs[, 98]+genet.bs[, 99]+genet.bs[, 100]+genet.bs[, 101]+genet.bs[, 102]+genet.bs[,
103]+genet.bs[, 104]+genet.bs[, 105]+genet.bs[, 106]+genet.bs[, 107]+genet.bs[, 108]+genet.bs[,
109]+genet.bs[, 110]+genet.bs[, 111]+genet.bs[, 112]+genet.bs[, 113]+genet.bs[, 114]+genet.bs[,
115]+genet.bs[, 116]+genet.bs[, 117]+genet.bs[, 118]+genet.bs[, 119]+genet.bs[, 120]+genet.bs[,
121]+genet.bs[, 122]+genet.bs[, 123]+genet.bs[, 124]+genet.bs[, 125]+genet.bs[, 126]+genet.bs[,
127]+genet.bs[, 128]+genet.bs[, 129]+genet.bs[, 130]+genet.bs[, 131]+genet.bs[, 132]+genet.bs[,
133]+genet.bs[, 134]+genet.bs[, 135]+genet.bs[, 136]+genet.bs[, 137]+genet.bs[, 138]+genet.bs[,
139]+genet.bs[, 140]+genet.bs[, 141]+genet.bs[, 142]+genet.bs[, 143]+genet.bs[, 144]+genet.bs[,
145]+genet.bs[, 146]+genet.bs[, 147]+genet.bs[, 148]+genet.bs[, 149]+genet.bs[, 150]+genet.bs[,
151]+genet.bs[, 152]+genet.bs[, 153]+genet.bs[, 154]+genet.bs[, 155]+genet.bs[, 156]+genet.bs[,
157]+genet.bs[, 158]+genet.bs[, 159]+genet.bs[, 160]+genet.bs[, 161]+genet.bs[, 162]+genet.bs[,
163]+genet.bs[, 164]+genet.bs[, 165]+genet.bs[, 166]+genet.bs[, 167]+genet.bs[, 168]+genet.bs[,
169]+genet.bs[, 170]+genet.bs[, 171]+genet.bs[, 172]+genet.bs[, 173]+genet.bs[, 174]+genet.bs[,
175]+genet.bs[, 176]+genet.bs[, 177]+genet.bs[, 178]+genet.bs[, 179]+genet.bs[, 180]+genet.bs[,
181]+genet.bs[, 182]+genet.bs[, 183]+genet.bs[, 184]+genet.bs[, 185]+genet.bs[, 186]+genet.bs[,
187]+genet.bs[, 188]+genet.bs[, 189]+genet.bs[, 190]+genet.bs[, 191]+genet.bs[, 192]+genet.bs[,
193]+genet.bs[, 194]+genet.bs[, 195]+genet.bs[, 196]+genet.bs[, 197]+genet.bs[, 198]+genet.bs[,
199]+genet.bs[, 200]+genet.bs[, 201]+genet.bs[, 202]+genet.bs[, 203]+genet.bs[, 204]+genet.bs[,
205]+genet.bs[, 206]+genet.bs[, 207]+genet.bs[, 208]+genet.bs[, 209]+genet.bs[, 210]+genet.bs[,
```

```
211]+genet.bs[, 212]+genet.bs[, 213]+genet.bs[, 214]+genet.bs[, 215]+genet.bs[, 216]+genet.bs[,
217]+genet.bs[, 218])


###ordistep for binary with all the PCs, here almost most of PCs are significant
gen_bin= ordistep(mod0, mod1, Pin=0.05, permutations=999)
save(gen_bin, file="ordistep_gen_bin.Rdata")
load("ordistep_gen_bin.Rdata")

### WITH 999 PERM, select the significant axis that been selected by ordistep
GENET=data.frame(cbind(genet.bs[, 151] ,genet.bs[, 126] ,genet.bs[, 28] ,genet.bs[, 42] ,genet.bs[,
178] ,genet.bs[, 35] ,genet.bs[, 102] ,genet.bs[, 166] ,genet.bs[, 149] ,genet.bs[, 209] ,genet.bs[, 135]
,genet.bs[, 95] ,genet.bs[, 6] ,genet.bs[, 156] ,genet.bs[, 73] ,genet.bs[, 158] ,genet.bs[, 23]
,genet.bs[, 133] ,genet.bs[, 21] ,genet.bs[, 199] ,genet.bs[, 183] ,genet.bs[, 39] ,genet.bs[, 5]
,genet.bs[, 116] ,genet.bs[, 177] ,genet.bs[, 186] ,genet.bs[, 86] ,genet.bs[, 88] ,genet.bs[, 90]
,genet.bs[, 34]))
#
mod2=rda(m_P~meth.bs[, 1]+meth.bs[, 2]+meth.bs[, 3]+meth.bs[, 4]+meth.bs[, 5]+meth.bs[,
6]+meth.bs[, 7]+meth.bs[, 8]+meth.bs[, 9]+meth.bs[, 10]+meth.bs[, 11]+meth.bs[, 12]+meth.bs[,
13]+meth.bs[, 14]+meth.bs[, 15]+meth.bs[, 16]+meth.bs[, 17]+meth.bs[, 18]+meth.bs[,
19]+meth.bs[, 20]+meth.bs[, 21]+meth.bs[, 22]+meth.bs[, 23]+meth.bs[, 24]+meth.bs[,
25]+meth.bs[, 26]+meth.bs[, 27]+meth.bs[, 28]+meth.bs[, 29]+meth.bs[, 30]+meth.bs[,
31]+meth.bs[, 32]+meth.bs[, 33]+meth.bs[, 34]+meth.bs[, 35]+meth.bs[, 36]+meth.bs[,
37]+meth.bs[, 38]+meth.bs[, 39]+meth.bs[, 40]+meth.bs[, 41]+meth.bs[, 42]+meth.bs[,
43]+meth.bs[, 44]+meth.bs[, 45]+meth.bs[, 46]+meth.bs[, 47]+meth.bs[, 48]+meth.bs[,
49]+meth.bs[, 50]+meth.bs[, 51]+meth.bs[, 52]+meth.bs[, 53]+meth.bs[, 54]+meth.bs[,
55]+meth.bs[, 56]+meth.bs[, 57]+meth.bs[, 58]+meth.bs[, 59]+meth.bs[, 60]+meth.bs[,
61]+meth.bs[, 62]+meth.bs[, 63]+meth.bs[, 64]+meth.bs[, 65]+meth.bs[, 66]+meth.bs[,
67]+meth.bs[, 68]+meth.bs[, 69]+meth.bs[, 70]+meth.bs[, 71]+meth.bs[, 72]+meth.bs[,
73]+meth.bs[, 74]+meth.bs[, 75]+meth.bs[, 76]+meth.bs[, 77]+meth.bs[, 78]+meth.bs[,
79]+meth.bs[, 80]+meth.bs[, 81]+meth.bs[, 82]+meth.bs[, 83]+meth.bs[, 84]+meth.bs[,
85]+meth.bs[, 86]+meth.bs[, 87]+meth.bs[, 88]+meth.bs[, 89]+meth.bs[, 90]+meth.bs[,
91]+meth.bs[, 92]+meth.bs[, 93]+meth.bs[, 94]+meth.bs[, 95]+meth.bs[, 96]+meth.bs[,
97]+meth.bs[, 98]+meth.bs[, 99]+meth.bs[, 100]+meth.bs[, 101]+meth.bs[, 102]+meth.bs[,
103]+meth.bs[, 104]+meth.bs[, 105]+meth.bs[, 106]+meth.bs[, 107]+meth.bs[, 108]+meth.bs[,
109]+meth.bs[, 110]+meth.bs[, 111]+meth.bs[, 112]+meth.bs[, 113]+meth.bs[, 114]+meth.bs[,
115]+meth.bs[, 116]+meth.bs[, 117]+meth.bs[, 118]+meth.bs[, 119]+meth.bs[, 120]+meth.bs[,
121]+meth.bs[, 122]+meth.bs[, 123]+meth.bs[, 124]+meth.bs[, 125]+meth.bs[, 126]+meth.bs[,
127]+meth.bs[, 128]+meth.bs[, 129]+meth.bs[, 130]+meth.bs[, 131]+meth.bs[, 132]+meth.bs[,
133]+meth.bs[, 134]+meth.bs[, 135]+meth.bs[, 136]+meth.bs[, 137]+meth.bs[, 138]+meth.bs[,
139]+meth.bs[, 140]+meth.bs[, 141]+meth.bs[, 142]+meth.bs[, 143]+meth.bs[, 144]+meth.bs[,
145]+meth.bs[, 146]+meth.bs[, 147]+meth.bs[, 148]+meth.bs[, 149]+meth.bs[, 150]+meth.bs[,
151]+meth.bs[, 152]+meth.bs[, 153]+meth.bs[, 154]+meth.bs[, 155]+meth.bs[, 156]+meth.bs[,
157]+meth.bs[, 158]+meth.bs[, 159]+meth.bs[, 160]+meth.bs[, 161]+meth.bs[, 162]+meth.bs[,
163]+meth.bs[, 164]+meth.bs[, 165]+meth.bs[, 166]+meth.bs[, 167]+meth.bs[, 168]+meth.bs[,
169]+meth.bs[, 170]+meth.bs[, 171]+meth.bs[, 172]+meth.bs[, 173]+meth.bs[, 174]+meth.bs[,
175]+meth.bs[, 176]+meth.bs[, 177]+meth.bs[, 178]+meth.bs[, 179]+meth.bs[, 180]+meth.bs[,
181]+meth.bs[, 182]+meth.bs[, 183]+meth.bs[, 184]+meth.bs[, 185]+meth.bs[, 186]+meth.bs[,
187]+meth.bs[, 188]+meth.bs[, 189]+meth.bs[, 190]+meth.bs[, 191]+meth.bs[, 192]+meth.bs[,
193]+meth.bs[, 194]+meth.bs[, 195]+meth.bs[, 196]+meth.bs[, 197]+meth.bs[, 198]+meth.bs[,
199]+meth.bs[, 200]+meth.bs[, 201]+meth.bs[, 202]+meth.bs[, 203]+meth.bs[, 204]+meth.bs[,
205]+meth.bs[, 206]+meth.bs[, 207]+meth.bs[, 208]+meth.bs[, 209]+meth.bs[, 210]+meth.bs[,
```

```
211]+meth.bs[, 212]+meth.bs[, 213]+meth.bs[, 214]+meth.bs[, 215]+meth.bs[, 216]+meth.bs[,
217]+meth.bs[, 218])
anova(mod2)
#
meth_bin= ordistep(mod0, mod2, Pin=0.05, permutations=999)
save(meth_bin, file="ordistep_meth_bin.Rdata")
load("ordistep_meth_bin.Rdata")

### WITH 999 PERM, select the significant axis that been selected by ordistep
METH=data.frame(cbind(meth.bs[, 2] ,meth.bs[, 6] ,meth.bs[, 4] ,meth.bs[, 35] ,meth.bs[, 24]
,meth.bs[, 1] ,meth.bs[, 72] ,meth.bs[, 15] ,meth.bs[, 100] ,meth.bs[, 106] ,meth.bs[, 86] ,meth.bs[,
26] ,meth.bs[, 77] ,meth.bs[, 22] ,meth.bs[, 145] ,meth.bs[, 208] ,meth.bs[, 141] ,meth.bs[, 200]
,meth.bs[, 5] ,meth.bs[, 43] ,meth.bs[, 32] ,meth.bs[, 13] ,meth.bs[, 158] ,meth.bs[, 197] ,meth.bs[,
81] ,meth.bs[, 108] ,meth.bs[, 153] ,meth.bs[, 20] ,meth.bs[, 121] ,meth.bs[, 148] ,meth.bs[, 55]
,meth.bs[, 149] ,meth.bs[, 103] ,meth.bs[, 90] ,meth.bs[, 94] ,meth.bs[, 129] ,meth.bs[, 82] ,meth.bs[,
83] ,meth.bs[, 68] ,meth.bs[, 87]))



varpart_bin = varpart(m_P,GENET,METH)
varpart_bin
# Partition of variance in RDA
#
# Call: varpart(Y = m_P, X = GENET, METH)
#
# Explanatory tables:
#   X1:  GENET
# X2:  METH
#
# No. of explanatory tables: 2
# Total variation (SS): 54.709
# Variance: 0.24981
# No. of observations: 220
#
# Partition table:
#   Df R.squared Adj.R.squared Testable
# [a+b] = X1          30   0.53922       0.46608     TRUE
# [b+c] = X2          40   0.66981       0.59602     TRUE
# [a+b+c] = X1+X2      70   0.81431       0.72707     TRUE
# Individual fractions
# [a] = X1|X2         30                0.13105     TRUE
# [b]                 0                0.33503    FALSE
# [c] = X2|X1         40                0.26098     TRUE
# [d] = Residuals                       0.27293    FALSE
# ---
#   Use function 'rda' to test significance of fractions of interest

plot(varpart_bin,  digits = 1, Xnames = c('Genetic', 'Epigenetic'), bg = c('Blue', 'red'))

################## With semi-continuous phenotype
#
modA=rda(m_P_con~1)
```

```
modB=rda(m_P_con~genet.bs[, 1]+genet.bs[, 2]+genet.bs[, 3]+genet.bs[, 4]+genet.bs[, 5]+genet.bs[,
6]+genet.bs[, 7]+genet.bs[, 8]+genet.bs[, 9]+genet.bs[, 10]+genet.bs[, 11]+genet.bs[, 12]+genet.bs[,
13]+genet.bs[, 14]+genet.bs[, 15]+genet.bs[, 16]+genet.bs[, 17]+genet.bs[, 18]+genet.bs[,
19]+genet.bs[, 20]+genet.bs[, 21]+genet.bs[, 22]+genet.bs[, 23]+genet.bs[, 24]+genet.bs[,
25]+genet.bs[, 26]+genet.bs[, 27]+genet.bs[, 28]+genet.bs[, 29]+genet.bs[, 30]+genet.bs[,
31]+genet.bs[, 32]+genet.bs[, 33]+genet.bs[, 34]+genet.bs[, 35]+genet.bs[, 36]+genet.bs[,
37]+genet.bs[, 38]+genet.bs[, 39]+genet.bs[, 40]+genet.bs[, 41]+genet.bs[, 42]+genet.bs[,
43]+genet.bs[, 44]+genet.bs[, 45]+genet.bs[, 46]+genet.bs[, 47]+genet.bs[, 48]+genet.bs[,
49]+genet.bs[, 50]+genet.bs[, 51]+genet.bs[, 52]+genet.bs[, 53]+genet.bs[, 54]+genet.bs[,
55]+genet.bs[, 56]+genet.bs[, 57]+genet.bs[, 58]+genet.bs[, 59]+genet.bs[, 60]+genet.bs[,
61]+genet.bs[, 62]+genet.bs[, 63]+genet.bs[, 64]+genet.bs[, 65]+genet.bs[, 66]+genet.bs[,
67]+genet.bs[, 68]+genet.bs[, 69]+genet.bs[, 70]+genet.bs[, 71]+genet.bs[, 72]+genet.bs[,
73]+genet.bs[, 74]+genet.bs[, 75]+genet.bs[, 76]+genet.bs[, 77]+genet.bs[, 78]+genet.bs[,
79]+genet.bs[, 80]+genet.bs[, 81]+genet.bs[, 82]+genet.bs[, 83]+genet.bs[, 84]+genet.bs[,
85]+genet.bs[, 86]+genet.bs[, 87]+genet.bs[, 88]+genet.bs[, 89]+genet.bs[, 90]+genet.bs[,
91]+genet.bs[, 92]+genet.bs[, 93]+genet.bs[, 94]+genet.bs[, 95]+genet.bs[, 96]+genet.bs[,
97]+genet.bs[, 98]+genet.bs[, 99]+genet.bs[, 100]+genet.bs[, 101]+genet.bs[, 102]+genet.bs[,
103]+genet.bs[, 104]+genet.bs[, 105]+genet.bs[, 106]+genet.bs[, 107]+genet.bs[, 108]+genet.bs[,
109]+genet.bs[, 110]+genet.bs[, 111]+genet.bs[, 112]+genet.bs[, 113]+genet.bs[, 114]+genet.bs[,
115]+genet.bs[, 116]+genet.bs[, 117]+genet.bs[, 118]+genet.bs[, 119]+genet.bs[, 120]+genet.bs[,
121]+genet.bs[, 122]+genet.bs[, 123]+genet.bs[, 124]+genet.bs[, 125]+genet.bs[, 126]+genet.bs[,
127]+genet.bs[, 128]+genet.bs[, 129]+genet.bs[, 130]+genet.bs[, 131]+genet.bs[, 132]+genet.bs[,
133]+genet.bs[, 134]+genet.bs[, 135]+genet.bs[, 136]+genet.bs[, 137]+genet.bs[, 138]+genet.bs[,
139]+genet.bs[, 140]+genet.bs[, 141]+genet.bs[, 142]+genet.bs[, 143]+genet.bs[, 144]+genet.bs[,
145]+genet.bs[, 146]+genet.bs[, 147]+genet.bs[, 148]+genet.bs[, 149]+genet.bs[, 150]+genet.bs[,
151]+genet.bs[, 152]+genet.bs[, 153]+genet.bs[, 154]+genet.bs[, 155]+genet.bs[, 156]+genet.bs[,
157]+genet.bs[, 158]+genet.bs[, 159]+genet.bs[, 160]+genet.bs[, 161]+genet.bs[, 162]+genet.bs[,
163]+genet.bs[, 164]+genet.bs[, 165]+genet.bs[, 166]+genet.bs[, 167]+genet.bs[, 168]+genet.bs[,
169]+genet.bs[, 170]+genet.bs[, 171]+genet.bs[, 172]+genet.bs[, 173]+genet.bs[, 174]+genet.bs[,
175]+genet.bs[, 176]+genet.bs[, 177]+genet.bs[, 178]+genet.bs[, 179]+genet.bs[, 180]+genet.bs[,
181]+genet.bs[, 182]+genet.bs[, 183]+genet.bs[, 184]+genet.bs[, 185]+genet.bs[, 186]+genet.bs[,
187]+genet.bs[, 188]+genet.bs[, 189]+genet.bs[, 190]+genet.bs[, 191]+genet.bs[, 192]+genet.bs[,
193]+genet.bs[, 194]+genet.bs[, 195]+genet.bs[, 196]+genet.bs[, 197]+genet.bs[, 198]+genet.bs[,
199]+genet.bs[, 200]+genet.bs[, 201]+genet.bs[, 202]+genet.bs[, 203]+genet.bs[, 204]+genet.bs[,
205]+genet.bs[, 206]+genet.bs[, 207]+genet.bs[, 208]+genet.bs[, 209]+genet.bs[, 210]+genet.bs[,
211]+genet.bs[, 212]+genet.bs[, 213]+genet.bs[, 214]+genet.bs[, 215]+genet.bs[, 216]+genet.bs[,
217]+genet.bs[, 218])
#
#
###ordistep for binary with all the PCs, here almost most of PCs are significant
genet_con = ordistep(modA, modB, Pin=0.05, permutations=999)
save(genet_con, file="ordistep_genet_con.Rdata")
genet_con$anova

### WITH 999 PERM, select the significant axis that been selected by ordistep
GENET2=data.frame(cbind(genet.bs[, 35] ,genet.bs[, 151] ,genet.bs[, 28] ,genet.bs[, 126] ,genet.bs[,
42] ,genet.bs[, 73] ,genet.bs[, 21] ,genet.bs[, 178] ,genet.bs[, 102] ,genet.bs[, 158] ,genet.bs[, 166]
,genet.bs[, 23] ,genet.bs[, 45] ,genet.bs[, 6] ,genet.bs[, 209] ,genet.bs[, 146] ,genet.bs[, 55]
,genet.bs[, 5] ,genet.bs[, 133] ,genet.bs[, 116] ,genet.bs[, 95] ,genet.bs[, 135] ,genet.bs[, 118]
,genet.bs[, 149] ,genet.bs[, 215] ,genet.bs[, 205] ,genet.bs[, 72] ,genet.bs[, 159] ,genet.bs[, 98]))

#
```

```
modC=rda(m_P_con~meth.bs[, 1]+meth.bs[, 2]+meth.bs[, 3]+meth.bs[, 4]+meth.bs[, 5]+meth.bs[,
6]+meth.bs[, 7]+meth.bs[, 8]+meth.bs[, 9]+meth.bs[, 10]+meth.bs[, 11]+meth.bs[, 12]+meth.bs[,
13]+meth.bs[, 14]+meth.bs[, 15]+meth.bs[, 16]+meth.bs[, 17]+meth.bs[, 18]+meth.bs[,
19]+meth.bs[, 20]+meth.bs[, 21]+meth.bs[, 22]+meth.bs[, 23]+meth.bs[, 24]+meth.bs[,
25]+meth.bs[, 26]+meth.bs[, 27]+meth.bs[, 28]+meth.bs[, 29]+meth.bs[, 30]+meth.bs[,
31]+meth.bs[, 32]+meth.bs[, 33]+meth.bs[, 34]+meth.bs[, 35]+meth.bs[, 36]+meth.bs[,
37]+meth.bs[, 38]+meth.bs[, 39]+meth.bs[, 40]+meth.bs[, 41]+meth.bs[, 42]+meth.bs[,
43]+meth.bs[, 44]+meth.bs[, 45]+meth.bs[, 46]+meth.bs[, 47]+meth.bs[, 48]+meth.bs[,
49]+meth.bs[, 50]+meth.bs[, 51]+meth.bs[, 52]+meth.bs[, 53]+meth.bs[, 54]+meth.bs[,
55]+meth.bs[, 56]+meth.bs[, 57]+meth.bs[, 58]+meth.bs[, 59]+meth.bs[, 60]+meth.bs[,
61]+meth.bs[, 62]+meth.bs[, 63]+meth.bs[, 64]+meth.bs[, 65]+meth.bs[, 66]+meth.bs[,
67]+meth.bs[, 68]+meth.bs[, 69]+meth.bs[, 70]+meth.bs[, 71]+meth.bs[, 72]+meth.bs[,
73]+meth.bs[, 74]+meth.bs[, 75]+meth.bs[, 76]+meth.bs[, 77]+meth.bs[, 78]+meth.bs[,
79]+meth.bs[, 80]+meth.bs[, 81]+meth.bs[, 82]+meth.bs[, 83]+meth.bs[, 84]+meth.bs[,
85]+meth.bs[, 86]+meth.bs[, 87]+meth.bs[, 88]+meth.bs[, 89]+meth.bs[, 90]+meth.bs[,
91]+meth.bs[, 92]+meth.bs[, 93]+meth.bs[, 94]+meth.bs[, 95]+meth.bs[, 96]+meth.bs[,
97]+meth.bs[, 98]+meth.bs[, 99]+meth.bs[, 100]+meth.bs[, 101]+meth.bs[, 102]+meth.bs[,
103]+meth.bs[, 104]+meth.bs[, 105]+meth.bs[, 106]+meth.bs[, 107]+meth.bs[, 108]+meth.bs[,
109]+meth.bs[, 110]+meth.bs[, 111]+meth.bs[, 112]+meth.bs[, 113]+meth.bs[, 114]+meth.bs[,
115]+meth.bs[, 116]+meth.bs[, 117]+meth.bs[, 118]+meth.bs[, 119]+meth.bs[, 120]+meth.bs[,
121]+meth.bs[, 122]+meth.bs[, 123]+meth.bs[, 124]+meth.bs[, 125]+meth.bs[, 126]+meth.bs[,
127]+meth.bs[, 128]+meth.bs[, 129]+meth.bs[, 130]+meth.bs[, 131]+meth.bs[, 132]+meth.bs[,
133]+meth.bs[, 134]+meth.bs[, 135]+meth.bs[, 136]+meth.bs[, 137]+meth.bs[, 138]+meth.bs[,
139]+meth.bs[, 140]+meth.bs[, 141]+meth.bs[, 142]+meth.bs[, 143]+meth.bs[, 144]+meth.bs[,
145]+meth.bs[, 146]+meth.bs[, 147]+meth.bs[, 148]+meth.bs[, 149]+meth.bs[, 150]+meth.bs[,
151]+meth.bs[, 152]+meth.bs[, 153]+meth.bs[, 154]+meth.bs[, 155]+meth.bs[, 156]+meth.bs[,
157]+meth.bs[, 158]+meth.bs[, 159]+meth.bs[, 160]+meth.bs[, 161]+meth.bs[, 162]+meth.bs[,
163]+meth.bs[, 164]+meth.bs[, 165]+meth.bs[, 166]+meth.bs[, 167]+meth.bs[, 168]+meth.bs[,
169]+meth.bs[, 170]+meth.bs[, 171]+meth.bs[, 172]+meth.bs[, 173]+meth.bs[, 174]+meth.bs[,
175]+meth.bs[, 176]+meth.bs[, 177]+meth.bs[, 178]+meth.bs[, 179]+meth.bs[, 180]+meth.bs[,
181]+meth.bs[, 182]+meth.bs[, 183]+meth.bs[, 184]+meth.bs[, 185]+meth.bs[, 186]+meth.bs[,
187]+meth.bs[, 188]+meth.bs[, 189]+meth.bs[, 190]+meth.bs[, 191]+meth.bs[, 192]+meth.bs[,
193]+meth.bs[, 194]+meth.bs[, 195]+meth.bs[, 196]+meth.bs[, 197]+meth.bs[, 198]+meth.bs[,
199]+meth.bs[, 200]+meth.bs[, 201]+meth.bs[, 202]+meth.bs[, 203]+meth.bs[, 204]+meth.bs[,
205]+meth.bs[, 206]+meth.bs[, 207]+meth.bs[, 208]+meth.bs[, 209]+meth.bs[, 210]+meth.bs[,
211]+meth.bs[, 212]+meth.bs[, 213]+meth.bs[, 214]+meth.bs[, 215]+meth.bs[, 216]+meth.bs[,
217]+meth.bs[, 218])
#
#
meth_con = ordistep(modA, modC, Pin=0.05, permutations=999)
save(meth_con, file="ordistep_meth_con.Rdata")
meth_con$anova

### WITH 999 PERM, select the significant axis that been selected by ordistep
METH2=data.frame(cbind(meth.bs[, 4] ,meth.bs[, 2] ,meth.bs[, 1] ,meth.bs[, 6] ,meth.bs[, 24]
,meth.bs[, 35] ,meth.bs[, 15] ,meth.bs[, 5] ,meth.bs[, 100] ,meth.bs[, 106] ,meth.bs[, 22] ,meth.bs[,
86] ,meth.bs[, 145] ,meth.bs[, 32] ,meth.bs[, 72] ,meth.bs[, 141] ,meth.bs[, 153] ,meth.bs[, 26]
,meth.bs[, 108] ,meth.bs[, 177] ,meth.bs[, 30] ,meth.bs[, 139] ,meth.bs[, 66] ,meth.bs[, 202]
,meth.bs[, 103] ,meth.bs[, 77] ,meth.bs[, 112] ,meth.bs[, 82] ,meth.bs[, 119]))

varpart_con = varpart(m_P_con,GENET2,METH2)
varpart_con
```

```
# Partition of variance in RDA
#
# Call: varpart(Y = m_P_con, X = GENET2, METH2)
#
# Explanatory tables:
#   X1:  GENET2
# X2:  METH2
#
# No. of explanatory tables: 2
# Total variation (SS): 2627146
# Variance: 11996
# No. of observations: 220
#
# Partition table:
#   Df R.squared Adj.R.squared Testable
# [a+b] = X1          29   0.55111       0.48259     TRUE
# [b+c] = X2          29   0.57945       0.51527     TRUE
# [a+b+c] = X1+X2      58   0.74700       0.65586     TRUE
# Individual fractions
# [a] = X1|X2         29                0.14059     TRUE
# [b]                 0               0.34200    FALSE
# [c] = X2|X1         29                0.17327     TRUE
# [d] = Residuals                       0.34414    FALSE
# ---
#   Use function 'rda' to test significance of fractions of interest

plot(varpart_con,  digits = 1, Xnames =
```

Video S1: Video showing the "behaviour" of a moribund or freshly died oyster.

Supplementary Data: Table summarizing all the data used for this publication

Data S1 Oysters phenotyping (donors and recipients) either in binary (0=dead as susceptible and 1=alive as resistant) or in the semi-quantitative (accumulation hours alive) trait.

Data S2 Stats of the exome-capture sequencing: raw reads (Paire end, PE), trimming, alignment (V9 oyster genome), bisulfite conversion efficiency, total SNPs and CpGs.

Data S3 Genome Wide Association (GWA) mapping showing the 113 significant SNPs associated with POMS in binary trait.

Data S4 Genome Wide Association (GWA) mapping showing the 112 significant SNPs associated with POMS in semiquantitative trait.

Data S5 Location in the genome (exon, intron or close to a gene) of the 186 non-redundant SNPs identified from binary and semi-quantitative traits.

Data S6 The 155 genes with at least one signifcant SNPs identified from binary and semi-quantitativen trait.

Data S7 The 184 significant Gene Ontology (GO-terms) participating in Biological Process (BP) from the 155 genes with at least one significant SNPs (Data S6).

Data S8 Epigenome Wide Association (EWA) mapping showing the 240 significant CpGs associated with POMS in binary trait.

Data S9 Epigenome Wide Association (EWA) mapping showing the 226 significant CpGs associated with POMS in semi-quantitative trait.

Data S10 Location in the genome (exon, intron or close to a gene) of the 305 non-redundant CpGs identified from binary and semi-quantitative traits.

Data S11 The 171 genes with at least one signifcant CpGs identified from binary and semi-quantitativen traits.

Data S12 The 158 significant Gene Ontology (GO-terms) participating in Biological Process (BP) from the 171 genes with at least one significant CpGs (Data S11).

Data S13The number of genes participating in the 240 Gene Ontology terms (GO-terms) identified in the Genome (GWA) and Epigenome Wide Association (EWA) mappings

Data S14 The 320 genes identified in the Genome (GWA) and Epigenome Wide Association (EWA) mappings that displayed at least one significant/suggestive SNP or one significant CpG

Data S15 The genomic regions covered by the probes (5’ and 3’ ends of each exon ± 100 base pairs) designed from the V9 reference *Crassostrea gigas* genome.

Data S16 The Gene Ontology (GO-terms) list of Molecular Function (F), Biological Process (P) and Cellular Component (C) identified in each gene of Crassotrea gigas genome.
